# Sertoli cell survival and barrier function are regulated by miR-181c/d-*Pafah1b1* axis during mammalian spermatogenesis

**DOI:** 10.1007/s00018-022-04521-w

**Published:** 2022-08-25

**Authors:** Yue Feng, Dake Chen, Tiansu Wang, Jiawei Zhou, Wenning Xu, Hao Xiong, Rong Bai, Shang Wu, Jialian Li, Fenge Li

**Affiliations:** 1 Key Laboratory of Pig Genetics and Breeding of Ministry of Agriculture and Key Laboratory of Agricultural Animal Genetics, Breeding and Reproduction of Ministry of Education, Huazhong Agricultural University, Wuhan, 430070 People’s Republic of China; 2 Institute of Animal Science and Veterinary Medicine, Hubei Academy of Agricultural Sciences, Wuhan, 430064 People’s Republic of China; 3 The Cooperative Innovation Center for Sustainable Pig Production, Wuhan, 430070 People’s Republic of China; 4 College of Animal Science and Technology, Huazhong Agricultural University, Wuhan, 430070 People’s Republic of China

**Keywords:** miR-181c/d, *Pafah1b1*, Sertoli cells, Blood-testis barrier, Ectoplasmic specialization, Tight junction, Spermatogenesis, Mammals

## Abstract

Sertoli cells contribute to the formation of the blood-testis barrier (BTB), which is necessary for normal spermatogenesis. Recently, microRNAs (miRNAs) have emerged as posttranscriptional regulatory elements in BTB function during spermatogenesis. Our previous study has shown that miR-181c or miR-181d (miR-181c/d) is highly expressed in testes from boars at 60 days old compared with at 180 days old. Herein, we found that overexpression of miR-181c/d via miR-181c/d mimics in murine Sertoli cells (SCs) or through injecting miR-181c/d-overexpressing lentivirus in murine testes perturbs BTB function by altering BTB-associated protein distribution at the Sertoli cell–cell interface and F-actin organization, but this in vivo perturbation disappears approximately 6 weeks after the final treatment. We also found that miR-181c/d represses Sertoli cell proliferation and promotes its apoptosis. Moreover, miR-181c/d regulates Sertoli cell survival and barrier function by targeting platelet-activating factor acetylhydrolase 1b regulatory subunit 1 (*Pafah1b1*) gene. Furthermore, miR-181c/d suppresses PAFAH1B1 expression, reduces the complex of PAFAH1B1 with IQ motif-containing GTPase activating protein 1, and inhibits CDC42/PAK1/LIMK1/Cofilin pathway which is required for F-actin stabilization. In total, our results reveal the regulatory axis of miR-181c/d-*Pafah1b1* in cell survival and barrier function of Sertoli cells and provide additional insights into miRNA functions in mammalian spermatogenesis.

**Supplementary Information:**

The online version contains supplementary material available at 10.1007/s00018-022-04521-w.

## Introduction

Sertoli cells provide structural support and nourishment to germ cells during mammalian spermatogenesis [1]. Spermatogenesis efficiency is determined by the Sertoli cell number which depends on the proliferative capacity of immature Sertoli cells [2, 3]. In addition, the blood-testis barrier (BTB, also known as the Sertoli cell barrier) is constituted by the basal ectoplasmic specialization (ES) and several junction proteins between adjacent Sertoli cells and physically divides the seminiferous tubules into basal and apical compartments [4]. BTB maintains a proper microenvironment for controlling the development and maturation of germ cells during spermatogenesis [5], thus disruption of BTB often leads to germ cell loss and male infertility [6].

MicroRNAs (miRNAs) are a class of small non-coding RNAs with vital roles in cell survival, differentiation, and blood-tissue barrier [7–9]. Our previous miRNA microarray data showed that miR-181c or miR-181d (miR-181c/d) is highly expressed in testes from sexually immature boars at 60 days old compared with sexually mature boars at 180 days old [10]. At sexually immature stage, Sertoli cells have proliferative capacity and the BTB is not yet fully formed; at the sexually mature stage, Sertoli cells no longer undergo cell proliferation and have formed a blood-testis barrier [11–14]. The miR-181c is found to disturb the blood–brain barrier and F-actin organization in brain blood vessel endothelial cells by downregulating its target gene 3-phosphoinositide-dependent protein kinase-1 [15]. On the other hand, miR-181c promotes apoptosis and inhibits proliferation of HCV-infected hepatocytes [16]. Analogously, miR-181d suppresses cell proliferation and metastasis of gastric cancer via the PI3K/AKT signaling pathway [17]. Recently, increasing attention has been paid to the role of miRNAs in male infertility, especially in male germ cell development and differentiation [18, 19]. However, whether these miRNAs control BTB function and further regulate mammalian spermatogenesis remains largely uninvestigated.

Platelet-activating factor acetylhydrolase 1B subunit 1 (PAFAH1B1) (also known as Lissencephaly-1 (LIS1)) contains an N-terminal Lish domain and seven WD40 repeats at the C-terminal [20, 21]. Immunohistochemical staining of mouse testicular tissues showed PAFAH1B1 is localized in spermatogenic cells and Sertoli cells [22], and single-cell RNA-sequencing data effectively validates that PAFAH1B1 is expressed in germ cells, Sertoli cells, Leydig cells, and other cells of pig [23] and mouse [24] testis. Deletion of *Pafah1b1* in mice results in the failure of spermatids to form acrosomes and germ cell apoptosis [25, 26]. Studies also provide evidence for the roles of *Pafah1b1* in cholangiocarcinoma cell proliferation [27] and germinal center B cell apoptosis [28]. Additionally, the absence of *Pafah1b1* leads to F-actin cytoskeleton re-organization by downregulating Cdc42/Rac1 activities in neurons [29].

To investigate the function of miR-181c/d in male fertility, we established a Sertoli cell barrier in vitro to mimic BTB function in vivo and injected LV-miR-181c/d into mouse testes to overexpress miR-181c/d levels in the testes. Furthermore, we revealed the regulatory mechanism of the miR-181c/d-*Pafah1b1* gene on Sertoli cell survival and barrier function in mice. These results add to our understanding of miR-181c/d in mammalian spermatogenesis.

## Materials and methods

### Mice

Male Kunming mice were purchased from the experimental animal center of Huazhong Agricultural University and housed in a controlled environment (temperature of 22 ± 2 °C, relative humidity of 50–60%, light/dark cycle of 12 h/12 h) with free access to food and water. All the animal procedures were approved by the Institutional Animal Care and Use Committee of Huazhong Agricultural University.

### Cell culture and transfection

Primary murine SCs were isolated and purified from 18- to 21-day-old mouse testes [30]. Murine SCs were cultured in DMEM/F12 (11320033, Gibco) supplemented with 10% fetal bovine serum (10099141C, Gibco), bovine insulin (5 μg/mL), human transferrin (5 μg/mL), and epidermal growth factor (2.5 ng/mL). The swine testicular (ST) cells (ATCC Cat# CRL-1746, RRID: CVCL_2204) that have been identified as immature Sertoli cells [31] were purchased from the Cell Bank of Wuhan University (Wuhan, China). The porcine ST cells were cultured in DMEM/High Glucose medium (SH30022.01, HyClone) supplemented with 10% fetal bovine serum (10099141C, Gibco) at 37 °C with 5% CO_2_.

The full-length *Pafah1b1* cDNAs of mouse (NM_013625.4) and pig (NM_214250.1) were cloned into *pcDNA3.1* vector using Trelief™ SoSoo Cloning Kit (TSV-S2, Tsingke Biotechnology). The miRNAs and siRNAs were designed and synthesized by GenePharma (Shanghai, China). The plasmids, miRNAs, or siRNAs were transfected into cells using Lipofectamine™ 3000 (L3000015, Invitrogen) or RNAiMAX (13778030, Invitrogen) transfection reagent. The oligo sequence information is listed in Supplementary Table 1.

### Intratesticular injection with lentivirus of miR-181c/d

Murine miR-181c/d precursor sequences (mmu-mir-181c/d in Supplementary Table 1) were cloned into lentiviral vectors (LV) that contain green fluorescence protein ZsGreen (Hanbio, Shanghai, China). For in vivo experiments, sexually immature male mice at age of 16 days were randomly divided into three groups (*n* = 12). Mice in Groups I, II, and III were injected intratesticularly with miR-181c overexpression lentivirus (abbreviated as “LV-miR-181c”), miR-181d overexpression lentivirus (abbreviated as “LV-miR-181d”), control lentivirus (abbreviated as “LV-control”), respectively. Mice were anesthetized with 5% chloral hydrate (0.5 ml/100 g body weight). The scrotum was shaved, and then washed with antiseptic soap and wiped with ethanol. At age of 16 and 30 days, mice were injected with lentiviral solution. Each testis was located and held in position by one person while another person injected the lentiviral solution through the skin and into the testes (10 μL and 20 μL per testis) using a 30-gauge needle as described previously [32, 33]. Two weeks after the final injection, the mice were sacrificed by cervical dislocation.

### Indexes and histology of testis and epididymis

Murine testes and epididymides were isolated and weighted (*n* = 5). Testes and caput epididymides were fixed with 4% paraformaldehyde for 24 h, dehydrated for paraffin embedding, and transversely sectioned (5 μm thickness). Paraffin sections were stained using haematoxylin and eosin. Finally, the slides were observed under a light microscope (Olympus BX53, Japan).

### Sperm count and morphological analysis

Sperms were isolated from cauda epididymis and suspended in 500 μL of TYH medium (M2050, Easycheck) for 30 min at 37 °C. The sperm counts were calculated using a cell counting plate. For sperm morphological analyses, cauda epididymal sperms were spread onto glass slides and stained with Giemsa (*n* = 5).

### Transmission electron microscopy (TEM)

The freshly isolated testes (*n* = 3) and murine SCs were immersed and transferred into fresh TEM fixative solution at 4 °C. And then the samples were fixed with 1% OsO_4_ in phosphate-buffered saline (PBS). After removing OsO_4_, the samples were washed three times with PBS. The ultrathin sections were mounted on copper grids and then double stained with 2% uranium acetate saturated alcohol solution and 2.6% lead citrate. The samples were examined with an 80 kV Transmission Electron Microscope (HT7800, Hitachi, Japan).

### Biotin tracer studies

The integrity of BTB was tested using a biotin tracer, as previously described [34]. Briefly, 2 weeks after the final administration, mice were anesthetized with 5% chloral hydrate (0.5 mL/100 g body weight) (*n* = 3). Thirty microliters of EZ-Link Sulfo-NHS-LC-Biotin solution (10 mg/mL in PBS) were injected into the testicular interstitium. After 30 min, the mice were euthanized. The testes were collected, embedded in Tissue-Tek O. C. T Compound (Sakura Finetek, Japan), and frozen at − 80 °C until use. Frozen sections (6 μm thickness) were fixed with 4% paraformaldehyde for 15 min and incubated with Streptavidin-FITC (S3762, Sigma–Aldrich). The cell nuclei were stained with 4’, 6-diamidino-2-phenylindole (DAPI) (D9542, Sigma–Aldrich). Fluorescence images were visualized using an epifluorescence microscope (Olympus BX53, Japan). CdCl_2_ is known to induce BTB disruption [35], and mice injected intraperitoneally with CdCl_2_ (1 mg/kg) continuously for three days were used as positive controls. Randomly selected fields from each testis tissue section were evaluated. To semi-quantify the extent of BTB damage, we measured the distance traveled by biotin in the tubule (D_Biotin_) and the radius of the same tubule (D_Radius_). For an oval-shaped tubule, the radius is the average of the shortest and the longest distance of the tubule. The extent of the BTB damage can be expressed in percentage as: *E* = [*D*_Biotin_/*D*_Radius_] × 100% [36]. The relative distance of fluorescence distribution was quantified using Image J software.

### Immunofluorescence and F-actin staining

Immunofluorescence staining was performed as previously described [37, 38]. Briefly, frozen sections of testes (*n* = 3) or freshly isolated murine SCs cultured on coverslips were fixed with 4% paraformaldehyde for 15 min and then washed with PBS. The samples were incubated with primary antibodies and secondary antibodies. Cell nuclei were stained with DAPI. The following antibodies were used: Ki67 (A2094, ABclonal; 1:100), N-cadherin (33-3900, Invitrogen; 1:100), Occludin (71–1500, Invitrogen; 1:100), PAFAH1B1 (sc-374586, Santa Cruz; 1:100), PLZF (sc-28319, Santa Cruz; 1:100), ZO-1 (61-7300, Invitrogen; 1:100), β-catenin (71-2700, Invitrogen; 1:100), WT1 (ab89901, Abcam; 1:50), FITC Goat Anti-Mouse IgG (F0257, Sigma; 1:200), FITC Goat Anti-Rabbit IgG (F0382, Sigma; 1:200), CY3 Goat Anti-Rabbit IgG (SA00009-2, Proteintech; 1:200), and CY3 Goat Anti-Mouse IgG (SA00009-1, Proteintech; 1:200). Randomly selected fields from each testis tissue section were evaluated. The relative distance of fluorescence distribution was quantified using Image J software.

For F-actin staining, testis sections (*n* = 3) or murine SCs were incubated with Alexa Fluor 594 phalloidin (A12381, Invitrogen) or Alexa Fluor 488 phalloidin (A12379, Invitrogen). Cell nuclei were stained with DAPI. Fluorescence images were visualized using an epifluorescence microscope (Olympus BX53, Japan) or a confocal laser scanning microscope (Zeiss LSM 800, Carl Zeiss Imaging, Germany).

### Assessment of the permeability of the Sertoli cell barrier in vitro

Murine SCs were plated on Matrigel-coated Millicell bicameral units (diameter, 12 mm; pore size, 0.45 μm; effective surface area, 0.33 cm^2^, Millipore Corp) in 24-well plates containing 0.5 mL F12/DMEM. The permeability of the Sertoli cell barrier can be assessed in vitro by quantifying the trans-epithelial resistance (TER) with the Millicell ERS system (Millipore Corp) [39]. TER value was measured at three different areas in each bicameral culture. TER values of each sample were calculated as TER_sample_ (Ω cm^2^) = (*R*_sample_ – *R*_blank_) (Ω) × effective membrane area (cm^2^).

The permeability of the Sertoli cell barrier was also assessed in vitro using sodium fluorescein (Na-F) [40]. The Na-F concentration in the basal chamber of the control group before treatment was arbitrarily set as 100% for the experiment.

### Cell Counting Kit-8 assay

The cell viability was assessed using Cell Counting Kit-8 (CCK-8; CK04, Dojindo). Ten microliters of CCK-8 reagent were added to each well and incubated at 37 °C for 2 h. The data of optical density value at 450 nm was measured by a microplate reader (Bio-Rad, USA).

### Cell apoptosis assays

Cell apoptosis analysis was performed using an Annexin V-FITC Apoptosis Detection Kit (AD10, Dojindo) with FACS Calibur Flow Cytometry (Beckman Coulter, Brea, USA). For testis sections (*n* = 3), apoptotic cells were detected using the TUNEL Apoptosis Assay Kit (C1086, Beyotime). The testis sections were incubated with TUNEL reaction mixture for 60 min at 37 °C, then washed with PBS. Cell nuclei were stained with DAPI. Fluorescence images were visualized using an epifluorescence microscope (Olympus BX53, Japan).

### Dual-luciferase reporter assay

The fragments of *Pafah1b1* 3’ untranslated region (3’ UTR) containing the wild-type or mutated miR-181c/d binding sites were amplified and cloned into the *pmirGLO* dual-luciferase vector (Promega). Primers used in the experiment are listed in Supplementary Table 1. The recombinant construct plasmids were co-transfected with miR-181c/d mimics or mimics NC into porcine ST cells and murine SCs. Luciferase activity was measured with the Dual-Luciferase Reporter Assay System (E1960, Promega). Firefly luciferase activity was normalized to Renilla luciferase activity for each sample.

### Real-time quantitative PCR (RT-qPCR)

Total RNA was extracted using the TRIzol™ Reagent (15596026, Invitrogen). RT-qPCR analysis was performed using the iTaq™ Universal SYBR^®^ Green Supermix (1725121, Bio-Rad) on a CFX384 Touch™ Real-Time PCR Detection System (Bio-Rad, USA). RT-qPCR primers are listed in Supplementary Table 1. U6 and β-actin were used as internal controls for the miR-181c/d and coding genes, respectively. The relative expression of miRNAs or genes was calculated using the 2^−△△*Ct*^ method.

### Western blot

Protein samples were transferred to polyvinylidene difluoride membrane (ISEQ00010, Millipore). The blots were blocked with 5% nonfat milk for 2 h and then incubated with primary antibodies and secondary antibodies. The Clarity Western ECL Substrate Kit (170-5061, Bio-rad) was used to visualize the immunoreactive bands. Images were captured with an Image Quant LAS4000 system (GE Healthcare Life Sciences, Piscataway, NJ, USA). β-actin served as a protein loading control. The following antibodies were used: BAX (A0207, ABclonal; 1:1000), BCL2 (60178-1-Ig, 1:3000; Proteintech,), CDC42 (ab187643, Abcam; 1:20000), Cofilin (A1704, ABclonal; 1:1000), IQGAP1 (sc-376021, Santa Cruz; 1:500), LIMK1 (ab108507, Abcam; 1:5000), N-cadherin (33-3900, Invitrogen; 1:500), Occludin (71–1500, Invitrogen; 1:500), PAK1 (A19608, ABclonal; 1:1000), PAFAH1B1 (ab109630, Abcam; 1:5000), PCNA (A12427, ABclonal; 1:1000), p-Cofilin (AP0178, ABclonal; 1:1000), ZO-1 (61-7300, Invitrogen; 1:500), β-actin (AC028, ABclonal; 1:100000), β-catenin (71-2700, Invitrogen; 1:500), HRP Goat Anti-Mouse IgG (AS003, ABclonal; 1:3000), and HRP Goat Anti-Rabbit IgG (AS014, ABclonal; 1:3000).

### Co-immunoprecipitation

Sixty microlitres of Protein G magnetic beads (1614023, Bio-Rad) were incubated with antibodies for 2 h at room temperature. Then, the protein extracts were added to the beads and incubated overnight at 4 °C with rotation. The beads were washed with 1 × PBST. The proteins bound to the beads were eluted in standard 1 × SDS buffer and heated at 90 °C for 10 min. Finally, proteins were electrophoresed on 10% SDS–polyacrylamide gels and transferred to polyvinylidene difluoride membrane for the immunoblot analysis. IQGAP1 (sc-376021, Santa Cruz; 1:50) and PAFAH1B1 (sc-374586, Santa Cruz; 1:50) were used as the precipitating antibodies.

### Bioinformatic analysis

The potential binding sites of miR-181c/d within *Pafah1b1* 3’ UTR were predicted by Targetscan (http://www.targetscan.org/) online software. The three-dimensional structure of PAFAH1B1 and IQGAP1 proteins was predicted by I-TASSER (https://zhanggroup.org//I-TASSER/). The protein–protein interaction was performed by the ZDOCK server (https://zdock.umassmed.edu/).

### Statistical analysis

All data are presented as the mean ± standard deviation (SD). At least three independent experiments were performed and quantified. A two-tailed Student’s *t-test* was used for comparison between two groups. *p* < 0.05 was considered statistically significant.

## Results

### miR-181c/d delivery in murine testes increases Sertoli cell apoptosis and perturbs BTB function

The miR-181c/d were significantly upregulated in 60 d porcine testes compared to 180 d porcine testes (Supplementary Fig. 1a), consistent with the microarray data [10]. And in mice, we also examined the expression of miR-181c/d in testicular tissues at different developmental stages and found that the expression of miR-181c/d was higher in younger mice compared with older mice (Supplementary Fig. 1b). To explore the role of miR-181c/d in testicular development and spermatogenesis in mammals, we successfully overexpressed miR-181c/d by direct intratesticular injection of lentivirus-delivered miR-181c/d (LV-miR-181c/d) in mice (Supplementary Figs. 1c-f). LV-miR-181c/d treated mice showed similarities in testis size and testis weight/body weight ratio with the LV-control mice (Supplementary Fig. 1 g, h). Additionally, we evaluated the quality of sperms collected from the cauda epididymides of LV-control and LV-miR-181c/d treated mice. Although sperm count was not statistically different (Supplementary Fig. 1i), the abnormal sperm rate (including abnormal sperm head and tail rate) increased in LV-miR-181c/d mice (Supplementary Fig. 1j–l). Haematoxylin and eosin-stained sections showed no histological abnormalities in the testis or epididymis from the LV-control and LV-miR-181c/d mice (Supplementary Fig. 1 m). In addition, we analyzed cell proliferation and apoptosis in the testes by Ki67 and TUNEL staining, respectively. Compared with the LV-control testes, the LV-miR-181c/d murine testes had no alteration in cell proliferation (Supplementary Fig. 1n, o) but had a significant increase in cell apoptosis (Supplementary Fig. 1p, q). Furthermore, we found that the number of TUNEL and WT1 double-positive Sertoli cells increased (Supplementary Fig. 1p, r), but the number of undifferentiated spermatogonia (PLZF-positive cells) remained essentially unchanged (data not shown) in the testes of the LV-miR-181c/d treated group. These findings indicate that LV-miR-181c/d administration in testes increases the abnormal sperm rate and Sertoli cell apoptosis.

**Fig. 1:**
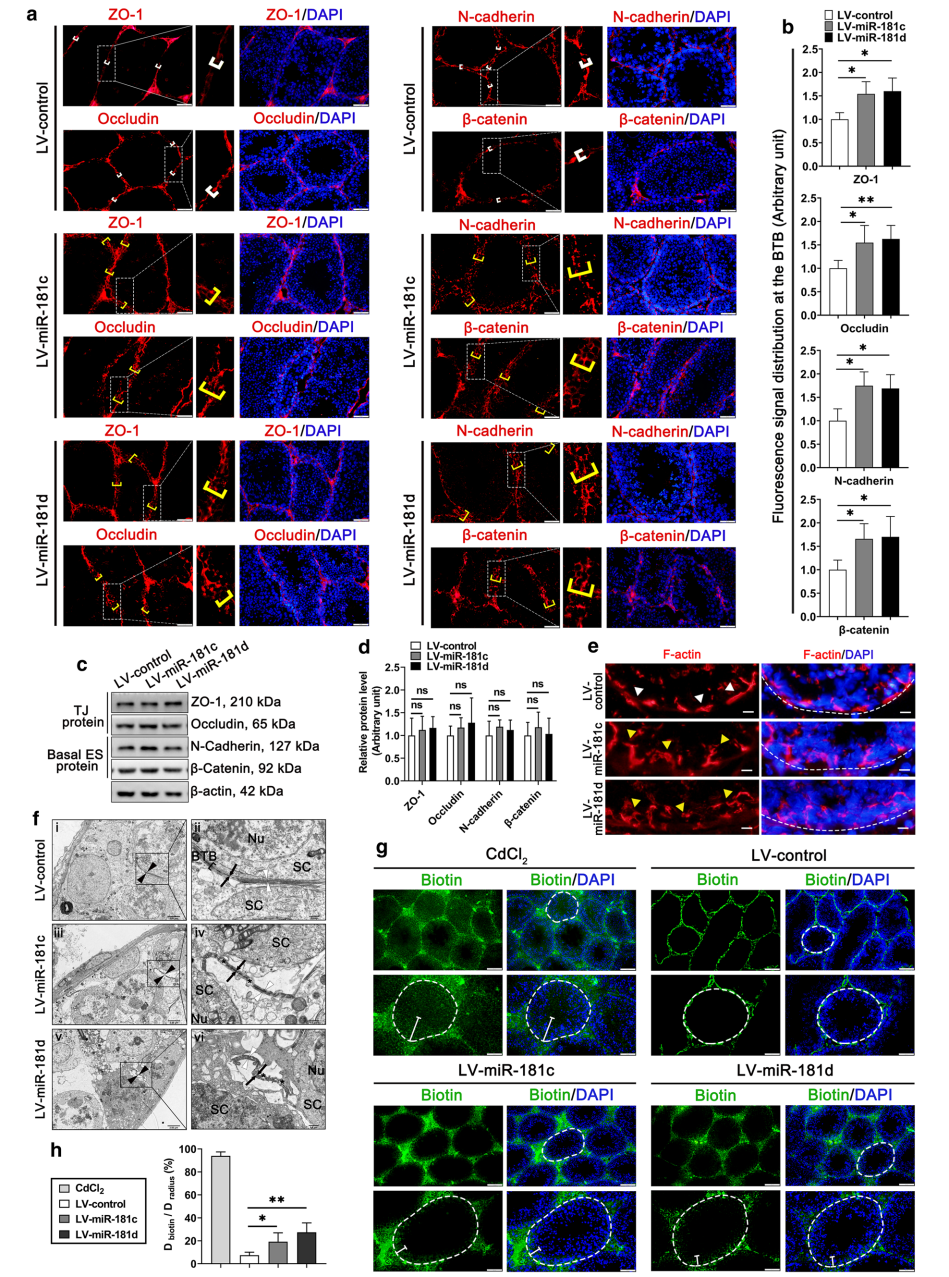
LV-miR-181c/d administration perturbs the BTB function in vivo*.* Mice were analyzed at 2 weeks post the final LV-miR-181c/d administration. **a** Immunofluorescence staining of TJ proteins (ZO-1, Occludin) (red) and basal ES proteins (N-cadherin, β-catenin) (red) in testes (*n* = 3). These proteins are tightly localized at the BTB (white brackets) or diffusely localized at the BTB (yellow brackets) near the basement membrane. Scale bars: 50 μm and 10 μm. **b** Quantification of fluorescence signal distributed at the BTB. **c** Western blot analysis of TJ proteins and basal ES proteins in testes. The quantification of protein level is shown in the bar graph (**d**). **e** F-actin staining (red) in mouse testis sections (*n* = 3). In LV-miR-181c/d mice, F-actin is no longer lined up properly along the BTB (yellow arrowheads) as found in the LV-control mice (white arrowheads). Scale bar: 10 μm. **f** TEM ultrastructural analysis of mouse testis (*n* = 3). Black arrowheads represent the interface of two SCs; black arrows represent the TJs structure. In the LV-control mice, white arrowheads represent the normal actin bundles. In LV-miR-181c/d mice, white arrowheads represent the dissolved actin bundles; asterisks represent the swollen intercellular space between adjacent SCs. Nu, nucleus; SC, Sertoli cell. Scale bars: 2.5 μm (**i**, **iii**, **v**), 0.5 μm (**ii**, **iv**, **vi**). **g** In vivo BTB integrity assay (*n* = 3). CdCl_2_-treated mice were used as positive controls. Disruption of the BTB is reflected by diffusion distance (white segments) of the indicator from the basal lamina (white broken circles) to the tubule lumen. Scale bars: 100 and 50 μm. **h** Histogram illustrating results of the BTB integrity assay. Data are presented as mean ± SD of at least three independent experiments. *p < 0.05; **p < 0.01; ns, not significant

The increase in abnormal sperm rate may be a consequence of a disrupted BTB structure [30, 41, 42]. Therefore, we detected whether miR-181c/d could affect the BTB function in vivo. Firstly, the distribution of tight junction (TJ) proteins (e.g., ZO-1, Occludin) and basal ES proteins (e.g., N-cadherin, β-catenin) at the BTB was perturbed in seminiferous tubules of LV-miR-181c/d treated mice (Fig. 1a, b). However, LV-miR-181c/d treatment failed to induce any remarkable changes in the expression level of multiple BTB-associated proteins (Fig. 1c, d). Furthermore, F-actin staining revealed that LV-miR-181c/d disturbed F-actin organization across the seminiferous epithelium (Fig. 1e). Results of LV-miR-181c/d-delivered testis ultrastructure examined by TEM showed there were intercellular spaces between adjacent murine SC contact at the BTB, coupled with TJ structure fractures (Fig. 1f). As indicated in Fig. 1g, h, LV-miR-181c/d administration in testes effectively disturbed BTB integrity, making biotin tracer penetrate the seminiferous tubules. This phenotype was similar to those in mice treated with CdCl_2_ (1 mg/kg) (Fig. 1g, h), which is well-known to induce BTB disruption [39]. Surprisingly, at 6 weeks post the final administration, the expression levels of miR-181c/d (data not shown) and the localization of BTB-associated proteins (Supplementary Fig. 2a, b) were virtually indistinguishable between the LV-control mice and the LV-miR-181c/d mice. Moreover, the damaged BTB integrity and the sperm quality were restored in LV-miR-181c/d mice (Supplementary Fig. 2c–e). The above results suggest that the in vivo LV-miR-181c/d treatment results in short-term BTB dysfunction in mice.

### miR-181c/d disturbs the Sertoli cell barrier by altering F-actin organization in vitro

**Fig. 2:**
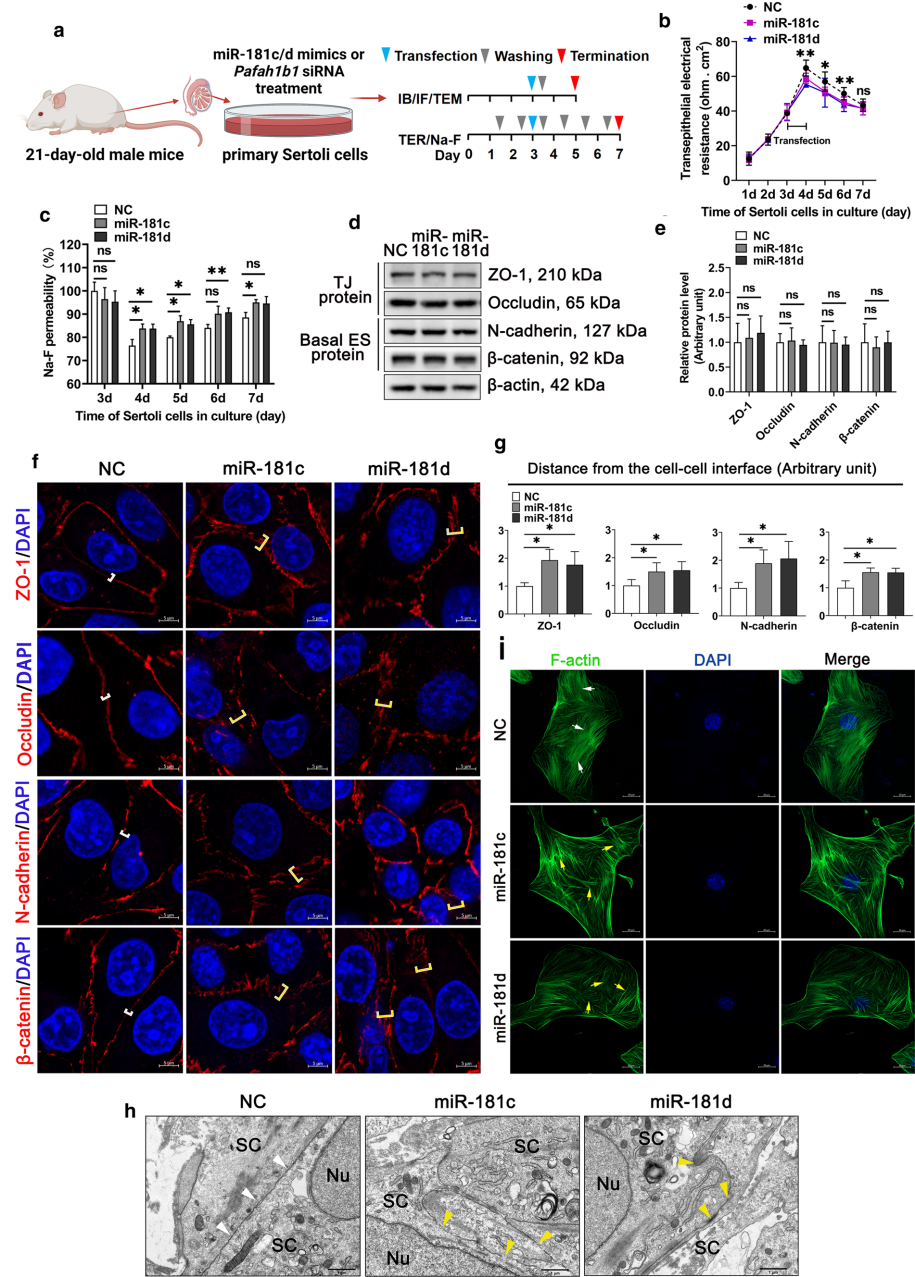
miR-181c/d overexpression disturbs the Sertoli cell barrier in vitro*.* Primary murine Sertoli cells (SCs) were transfected with mimics NC or miR-181c/d mimics. miR-181c mimics, miR-181d mimics, and mimics NC are abbreviated to miR-181c, miR-181d, and NC, respectively. **a** Schematic illustration of the treatment regimen. **b, c** The permeability of the Sertoli cell barrier was assessed in vitro by quantifying TER (**b**) or measuring the permeability of Na-F (**c**) in miR-181c/d mimics treated murine SCs. **d** Western blot analysis of TJ proteins and basal ES proteins in miR-181c/d mimics treated murine SCs. The quantification of protein level is shown in the bar graph (**e**). **f** Immunofluorescence staining of TJ proteins (red) and basal ES proteins (red) in miR-181c/d mimics treated murine SCs. These proteins are tightly localized (white brackets) or diffusively localized (yellow brackets) at the Sertoli cell–cell interface. Scale bar: 5 μm. **g** Quantification of fluorescence signal distributed at the cell–cell interface. **h** TEM ultrastructural analysis in miR-181c/d mimics treated murine SCs. Intact (white arrowheads) or disrupted (yellow arrowheads) TJ structures between adjacent murine SC contact. Scale bar: 1 μm. Nu, nucleus; SC, Sertoli cell. **i** F-actin staining (green) in miR-181c/d mimics treated murine SCs. Ordered (white arrows) or disordered (yellow arrows) F-actin are indicated. Scale bar: 20 μm. Data are presented as mean ± SD of at least three independent experiments. *p < 0.05; **p < 0.01; ns, not significant

Primary murine SCs cultured in vitro for 2–3 days can establish a functional TJ permeability barrier that mimics the BTB in vivo [43]. Overexpression of miR-181c/d in murine SCs resulted in a decreased TER value and an increased Na-F permeability (Fig. 2a–c), indicating miR-181c/d disturbs the Sertoli cell barrier integrity. Furthermore, even though the levels of TJ proteins and basal ES proteins remained unchanged (Fig. 2d, e), the distributions of TJ proteins and basal ES proteins at the Sertoli cell–cell interface were disturbed in miR-181c/d mimics treated murine SCs (Fig. 2f, g). According to the TEM results, overexpression of miR-181c/d led to several breaks and vacuoles at cell–cell contact (Fig. 2h), consistent with in vivo findings shown in Fig. 1f. These results indicate that miR-181c/d disturbs the Sertoli cell barrier, which may be mediated by changing the distribution of BTB-associated proteins at the Sertoli cell–cell interface. Since the actin-based cytoskeletons in Sertoli cells can support the attachment sites of TJ proteins and basal ES proteins [44], we then examined F-actin organization in murine SCs. As shown in Fig. 2i, F-actin was well-arranged and evenly distributed in the cytoplasm of control cells, but it was irregularly arranged, crossed, and no longer evenly distributed in the cytoplasm of murine SCs transfected with miR-181c/d mimics. These changes in F-actin organization thus contribute to altering the localization of TJ proteins and basal ES proteins, destabilizing cell junctions at the Sertoli cell–cell interface, and ultimately perturbing the Sertoli cell barrier.

### miR-181c/d inhibits Sertoli cell survival in vitro

**Fig. 3:**
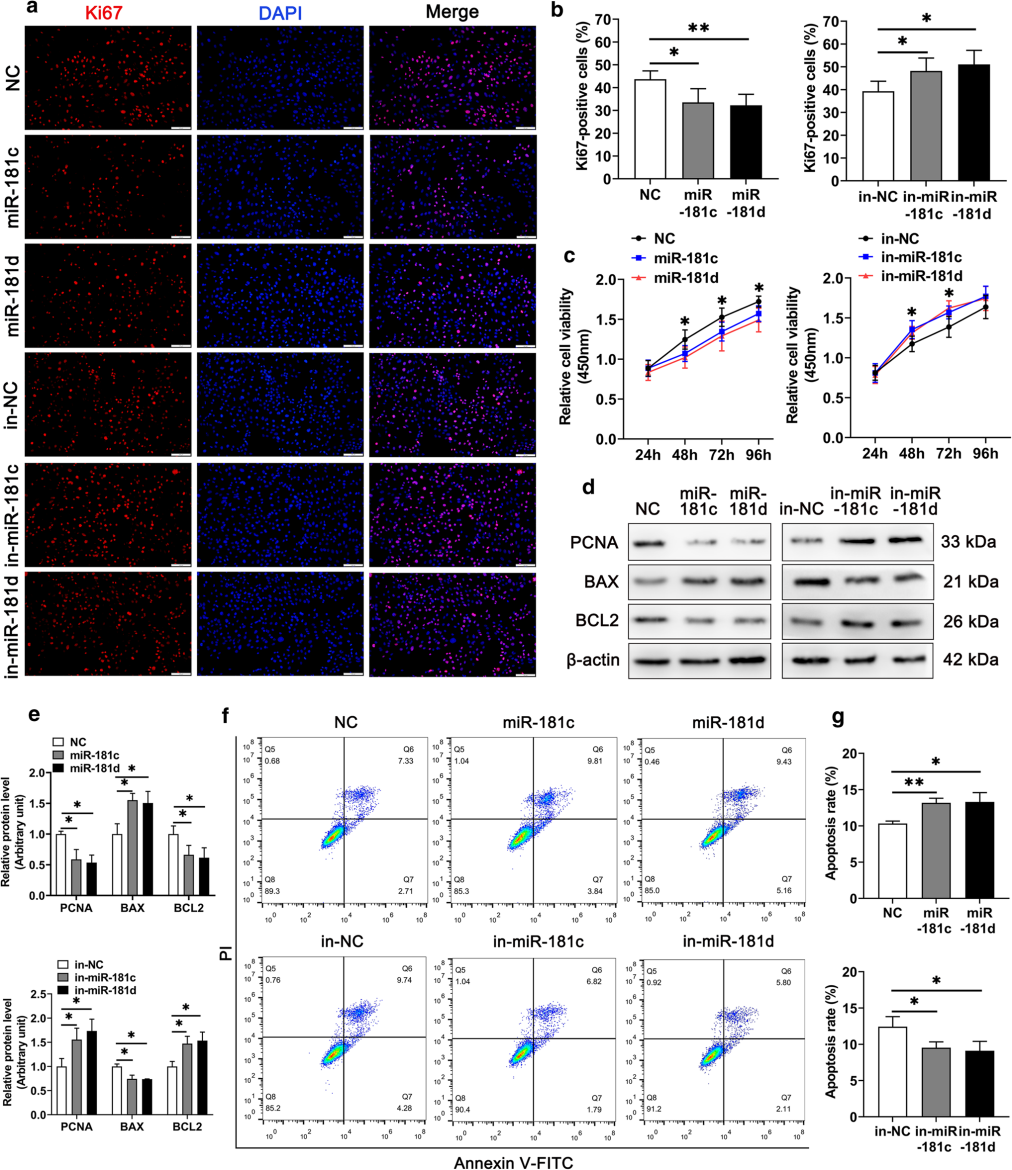
miR-181c/d inhibits proliferation and promotes apoptosis of murine Sertoli cells. The murine SCs were transfected with mimics NC, miR-181c/d mimics, inhibitors NC, or miR-181c/d inhibitors. miR-181c inhibitors, miR-181d inhibitors, and inhibitors NC are abbreviated to in-miR-181c, in-miR-181d, and in-NC, respectively. **a** Immunofluorescence staining of the cell proliferation marker Ki67 (red) in miR-181c/d mimics or inhibitors treated murine SCs. Scale bar: 100 µm. **b** Quantification of Ki67-positive cells in miR-181c/d mimics or inhibitors treated murine SCs. **c** CCK-8 assay performed in miR-181c/d mimics or inhibitors treated murine SCs. **d** Western blot analysis of PCNA, BAX, and BCL2 in miR-181c/d mimics or inhibitors treated murine SCs. The quantification of protein level is shown in the bar graph (**e**). **f** Annexin V-FITC/PI and flow cytometry analysis was used to examine cell apoptotic rate in miR-181c/d mimics or inhibitors treated murine SCs. **g** Quantification of cell apoptotic rate in miR-181c/d mimics or inhibitors treated murine SCs. Data are presented as mean ± SD of at least three independent experiments. *p < 0.05; **p < 0.01

It has been reported that BTB function disruption may be due in part to poor survival of Sertoli cells [45, 46], therefore, we examined the effects of miR-181c/d on cell survival in two types of Sertoli cells including primary murine Sertoli cells (SCs) and commercial swine testicular (ST) cells (immature Sertoli cells). The results of Ki67 staining and CCK-8 assay exhibited that overexpression of miR-181c/d significantly inhibited Sertoli cell proliferation (Fig. 3a–c and Supplementary Figs. 3a–c). Furthermore, overexpression of miR-181c/d reduced the levels of the proliferation marker proliferating cell nuclear antigen (PCNA) and anti-apoptotic B-cell lymphoma 2 (BCL2) but increased the level of the pro-apoptotic Bcl-2 associated X protein (BAX) (Fig. 3d, e and Supplementary Figs. 3d, e). Annexin V-FITC/PI and flow cytometry demonstrated that miR-181c/d increased the cell apoptotic rate in Sertoli cells (Fig. 3f, g and Supplementary Figs. 3f, g). Conversely, suppression of miR-181c/d repressed apoptosis and induced cell proliferation in Sertoli cells (Fig. 3a–g and Supplementary Fig. 3a–g). Above results demonstrate miR-181c/d increases apoptosis and inhibits proliferation in Sertoli cells.

### Knockdown of *Pafah1b1* disturbs the Sertoli cell barrier by changing F-actin organization in vitro

miRNA exerts its function through regulating its target genes, then we predicted the potential target gene of miR-181c/d using TargetScan online software (Supplementary Fig. 4a). The targeted sequences to the seed regions of miR-181c/d within the *Pafah1b1* 3’ UTR are conserved across species (Supplementary Fig. 4b). Subsequently, overexpression of miR-181c/d significantly repressed the luciferase activity of wild-type *Pafah1b1* 3’ UTR, but did not affect luciferase activity of mutated *Pafah1b1* 3’ UTR in murine SCs (Supplementary Figs. 4c, d) and porcine ST cells (Supplementary Fig. 4e, f). Furthermore, PAFAH1B1 protein level but not mRNA level was reduced in miR-181c/d overexpressed Sertoli cells (Supplementary Figs. 4 g–n) and murine testes (Supplementary Fig. 4o). Porcine *PAFAH1B1* transcripts are highly expressed in testes (Supplementary Fig. 4p, q). The expression levels of *Pafah1b1* in immature testes were significantly lower than in mature testes (Supplementary Figs. 4r–t), suggesting *Pafah1b1* had an opposite expression pattern to that of miR-181c/d in both pigs and mice (Supplementary Fig. 1a, b). Considered together, the *Pafah1b1* gene is one target of miR-181c/d.

**Fig. 4:**
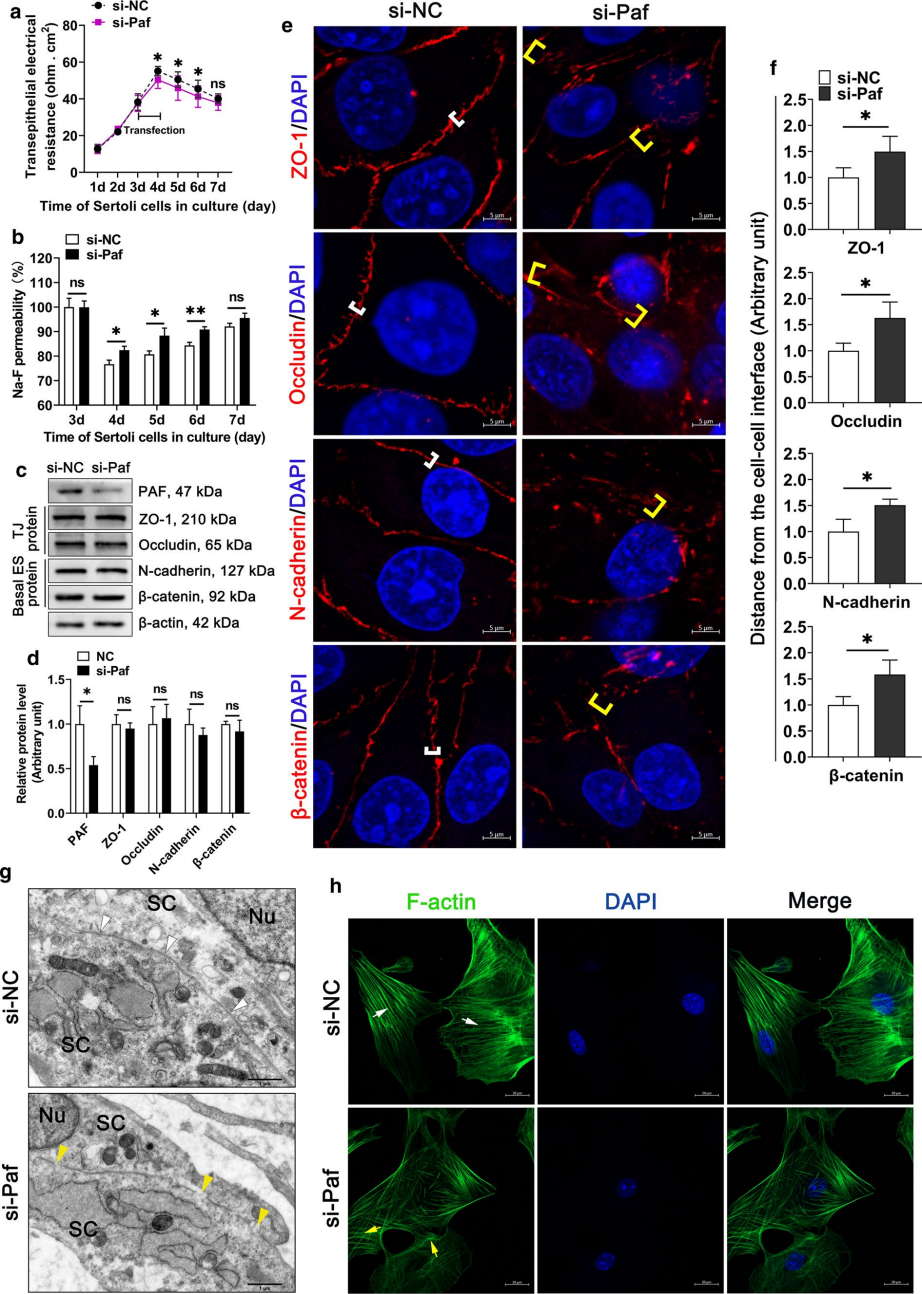
Inhibition of *Pafah1b1* disturbs the Sertoli cell barrier in vitro*.* The murine SCs were transfected with NC siRNA or *Pafah1b1* siRNA. NC siRNA and *Pafah1b1* siRNA are abbreviated to si-NC and si-*paf*, respectively. **a**, **b** The permeability of the Sertoli cell barrier was assessed in vitro by quantifying TER (**a**) or measuring the permeability of Na-F (**b**) in *Pafah1b1* siRNA treated murine SCs. **c** Western blot analysis of TJ proteins and basal ES proteins in *Pafah1b1* siRNA treated murine SCs. The quantification of protein level is shown in the bar graph (**d**). **e** Immunofluorescence staining of TJ proteins (red) and basal ES proteins (red) in *Pafah1b1* siRNA treated murine SCs. These proteins are tightly localized (white brackets) or diffusively localized (yellow brackets) at the Sertoli cell–cell interface. Scale bar: 5 μm. **f** Quantification of fluorescence signal distributed at the cell–cell interface. **g** TEM ultrastructural analysis in *Pafah1b1* siRNA treated murine SCs. Intact (white arrowheads) or disrupted (yellow arrowheads) TJ structures between adjacent murine SC contact. Scale bar: 1 μm. Nu, nucleus; SC, Sertoli cell. **h** F-actin staining (green) in *Pafah1b1* siRNA treated murine SCs. Ordered (white arrows) or disordered (yellow arrows) F-actin are indicated. Scale bar: 20 μm. Data are presented as mean ± SD of at least three independent experiments. *p < 0.05; **p < 0.01; ns, not significant

Next, we examined whether knockdown of *Pafah1b1* could perturb the Sertoli cell barrier, analogous to the miR-181c/d mimics treatment. We observed the downregulated TER value (Fig. 4a) and the increased Na-F permeability (Fig. 4b) in murine SCs transfected with *Pafah1b1* siRNA. Even though the levels of BTB-associated proteins remained unchanged (Fig. 4c, d), the localization of TJ proteins and basal ES proteins became disorganized in *Pafah1b1* siRNA transfected murine SCs (Fig. 4e, f). TEM analysis revealed that knockdown of *Pafah1b1* led to some fractures and vacuoles of TJ structures between adjacent murine SC contact (Fig. 4g). Furthermore, phalloidin staining results showed *Pafah1b1* knockdown disturbed the organization of F-actin (Fig. 4h), which was consistent with results in miR-181c/d mimics treated murine SCs (Fig. 2i). The above results demonstrate that knockdown of *Pafah1b1* leads to alterations in F-actin organization, which may be responsible for the perturbation of the Sertoli cell barrier.

### miR-181c/d inhibits survival of Sertoli cells by targeting *Pafah1b1* gene

**Fig. 5:**
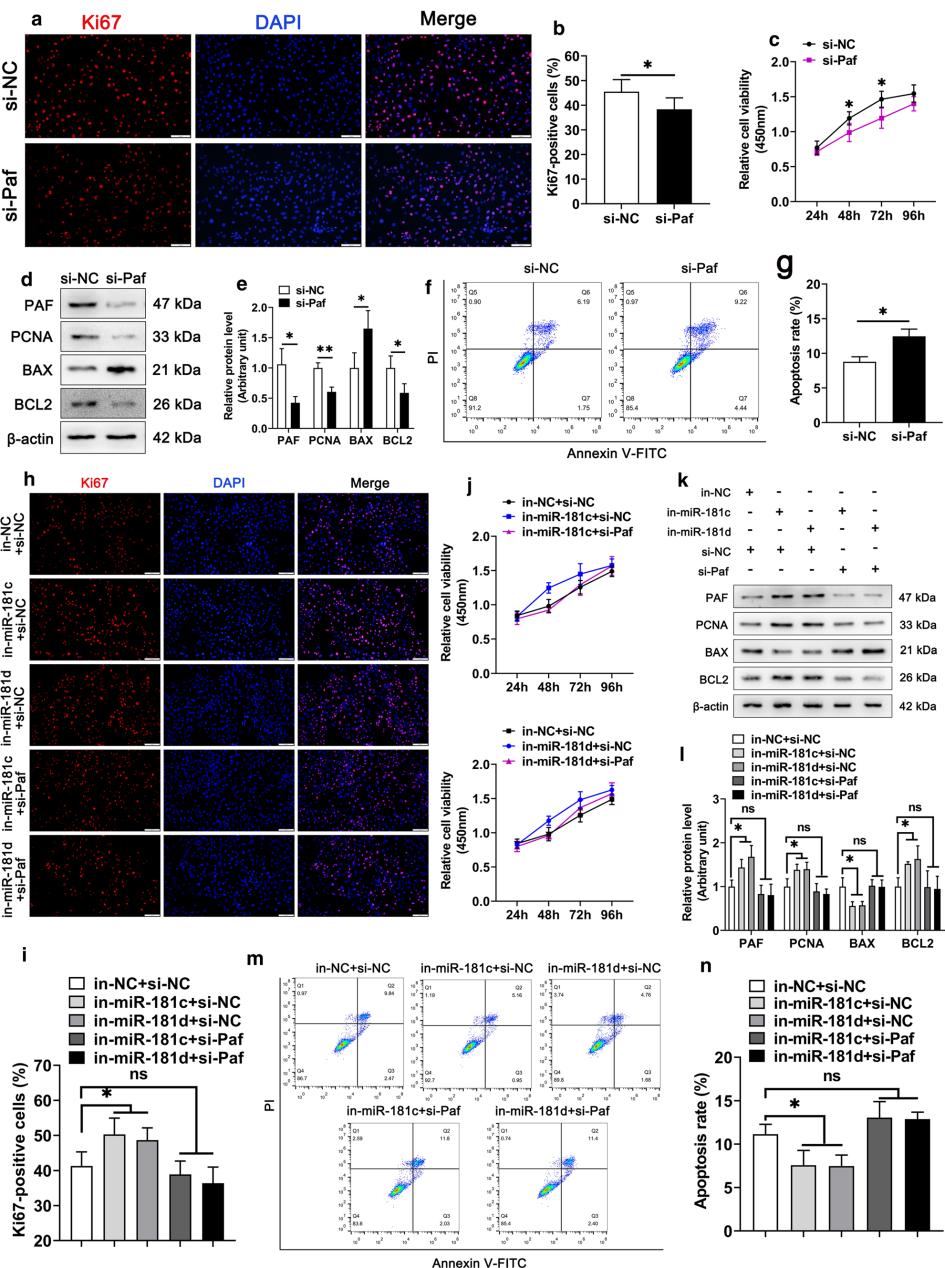
*Pafah1b1* knockdown reverses the pro-growth of miR-181c/d inhibited murine Sertoli cells. The murine SCs were transfected with NC siRNA or *Pafah1b1* siRNA. **a** Immunofluorescence staining of Ki67 (red) in *Pafah1b1* siRNA treated murine SCs. Scale bar: 100 µm. **b** Quantification of Ki67-positive cells in *Pafah1b1* siRNA treated murine SCs. **c** CCK-8 assay performed in *Pafah1b1* siRNA treated murine SCs. **d** Western blot analysis of PAFAH1B1, PCNA, BAX, and BCL2 in *Pafah1b1* siRNA treated murine SCs. The quantification of protein level is shown in the bar graph (**e**). **f** Annexin V-FITC/PI and flow cytometry analysis was used to examine cell apoptotic rate in *Pafah1b1* siRNA treated murine SCs. **g** The quantification of cell apoptotic rate in *Pafah1b1* siRNA treated murine SCs. Five co-transfection treatments were constructed in this experiment, including inhibitors NC + NC siRNA, miR-181c inhibitors + NC siRNA, miR-181d inhibitors + NC siRNA, miR-181c inhibitors + *Pafah1b1* siRNA, and miR-181d inhibitors + *Pafah1b1* siRNA. **h-j** Ki67 staining (**h**) and CCK-8 (**j**) assay were performed in murine SCs treated with co-transfections. Quantification of Ki67-positive murine SCs treated with co-transfections (**i**). Scale bar: 100 µm. **k** Western blot analysis of PAFAH1B1, PCNA, BAX, and BCL2 in murine SCs treated with co-transfections. The quantification of protein level is shown in the bar graph (**l**). **m** Annexin V-FITC/PI and flow cytometry analysis was used to examine cell apoptotic rate in murine SCs treated with co-transfections. **n** The quantification of cell apoptotic rate in murine SCs treated with co-transfections. Data are presented as mean ± SD of at least three independent experiments. *p < 0.05; **p < 0.01; ns, not significant

Given that inhibition of *Pafah1b1* had the similar regulatory function on the Sertoli cell barrier function with miR-181c/d overexpression, we speculated that *Pafah1b1* could also affect Sertoli cell survival. According to Ki67 staining and CCK-8 assay results, *Pafah1b1* silencing significantly inhibited Sertoli cell proliferation (Fig. 5a–c and Supplementary Figs. 5a–c). In addition, *Pafah1b1* silencing upregulated BAX expression and downregulated the levels of PCNA and BCL2 in Sertoli cells (Fig. 5d, e and Supplementary Figs. 5d, e). The Annexin V-FITC/PI and flow cytometry assay demonstrated that knockdown of *Pafah1b1* increased the cell apoptotic rate in Sertoli cells (Figs. 5f, g and Supplementary Figs. 5f, g). Conversely, overexpression of *Pafah1b1* increased Sertoli cell proliferation and decreased apoptosis (Supplementary Fig. 6a–n), indicating that *Pafah1b1* promotes proliferation and inhibits apoptosis of Sertoli cells.

Our present data suggest miR-181c/d inhibits proliferation and promotes apoptosis of Sertoli cells and *Pafah1b1* is a direct target of miR-181c/d. To detect whether miR-181c/d regulated Sertoli cell proliferation and apoptosis by targeting *Pafah1b1* gene, we assessed the proliferative and apoptotic phenotypes in Sertoli cells co-transfected with miR-181c/d inhibitors and *Pafah1b1* siRNA. We found that knockdown of *Pafah1b1* partially suppressed cell proliferation induced by miR-181c/d inhibitors (Fig. 5h–l and Supplementary Fig. 5 h–l). Accordingly, *Pafah1b1* silencing antagonized the inhibition effects of miR-181c/d inhibitors on cell apoptosis (Fig. 5m, n and Supplementary Fig. 5 m, n). These results demonstrate that miR-181c/d affects Sertoli cell proliferation and apoptosis by targeting the *Pafah1b1* gene.

### *Pafah1b1* promotes F-actin organization by interacting with IQGAP1

**Fig. 6:**
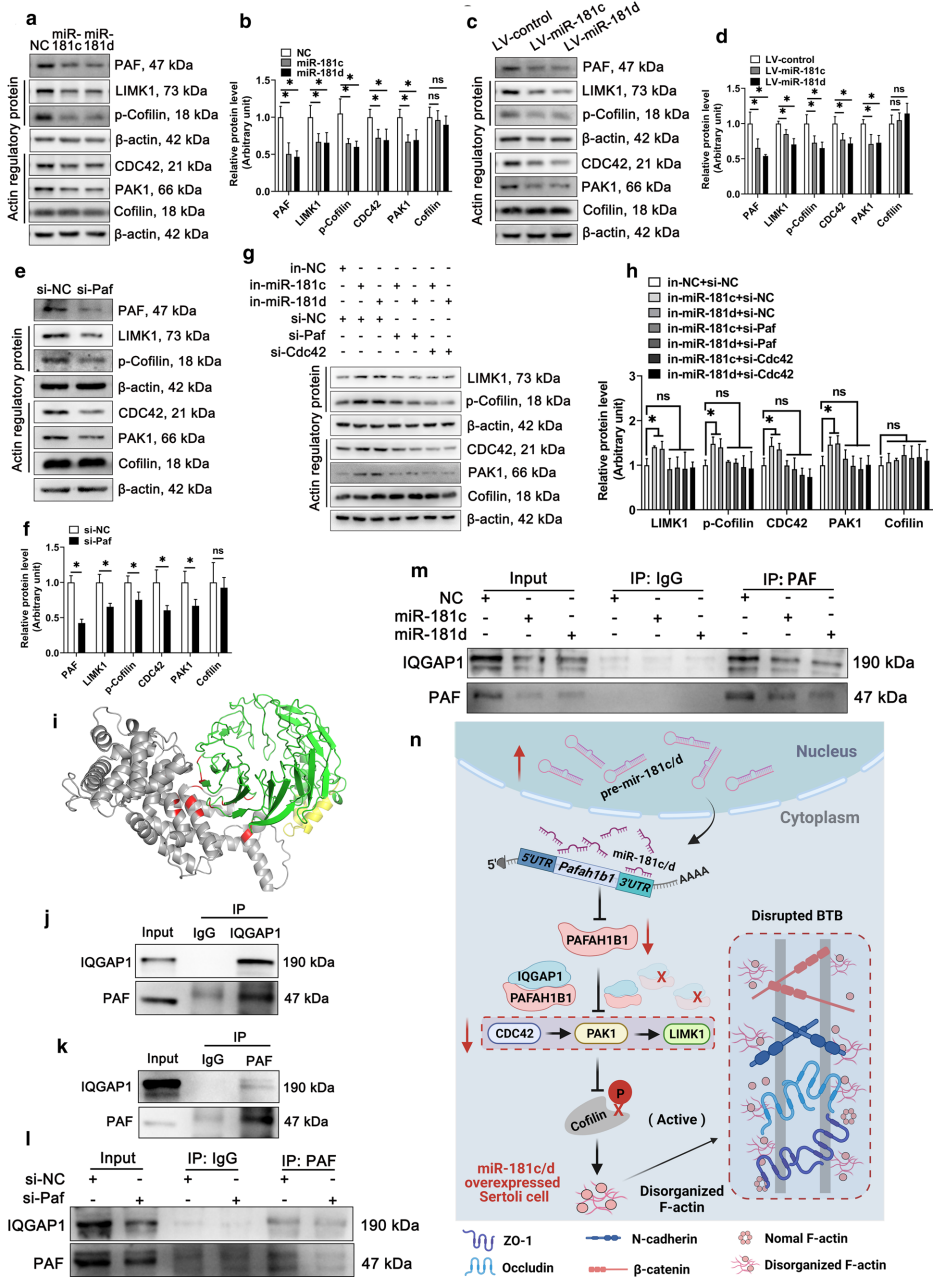
PAFAH1B1 regulates the expression of actin-regulatory proteins by interacting with IQGAP1 in murine Sertoli cells. **a-h** Western blot analysis and quantification for CDC42, PAK1, LIMK1, Cofilin, and p-Cofilin proteins in miR-181c/d mimics treated murine SCs (**a, b**), LV-miR-181c/d treated murine testes (**c, d**), *Pafah1b1* siRNA treated murine SCs (**e, f**), and miR-181c/d inhibitors + *Pafah1b1* siRNA or miR-181c/d inhibitors + *Cdc42* siRNA co-treated murine SCs (**g**, **h**). **i** The interaction between PAFAH1B1 and IQGAP1 was predicted by the ZDOCK server. PAFAH1B1 is indicated in green plus yellow; IQGAP1 is indicated in gray; the region of interaction is indicated in red. **j**, **k** Co-immunoprecipitation of PAFAH1B1 with IQGAP1 in murine SCs. **l**, **m** Immunoprecipitation assay was performed in *Pafah1b1* siRNA (**l**) or miR-181c/d mimics (**m**) treated murine SCs. **n** A schematic diagram illustrating the regulatory roles of the miR-181c/d-PAFAH1B1 axis on the Sertoli cell barrier function. Overexpression of miR-181c/d in murine SCs inhibits PAFAH1B1, which reduces the PAFAH1B1-IQGAP1 complex, resulting in the downregulation of CDC42 and its downstream PAK1, LIMK1, and p-Cofilin. Thus, changes in the expression of these actin-regulatory proteins impede the organization of F-actin, which could be a potential reason for perturbing the stabilization of the attachment sites between BTB-associated proteins and F-actin and disturbing the Sertoli cell barrier function

Overexpression of miR-181c/d or inhibition of *Pafah1b1* perturbed F-actin organization (Figs. 1e, 2i, and 4h), partly by altering the levels of actin-regulatory proteins that are important for F-actin cytoskeleton stability [47]. One of the actin-regulatory proteins, the cell division control protein 42 homolog (CDC42), can activate the p21(RAC1) activated kinase 1 (PAK1) and LIM domain kinase 1 (LIMK1). CDC42/PAK1/LIMK1 pathway leads to phosphorylation and inactivation of actin-regulatory protein Cofilin, and thus regulates F-actin cytoskeleton dynamics [48, 49]. Here, the levels of CDC42, PAK1, LIMK1, and p-Cofilin decreased not only in miR-181c/d overexpressed murine SCs (Fig. 6a, b) and testes (Fig. 6c, d), but also in *Pafah1b1* inhibited murine SCs (Fig. 6e, f). And knockdown of miR-181c/d increased actin-regulatory proteins expression, whereas transfection of *Pafah1b1* siRNA or *Cdc42* siRNA partially restored the elevated actin-regulatory proteins levels (Fig. 6g, h). These results indicate that the inactivation of the CDC42/PAK1/LIMK1/Cofilin pathway may be responsible for the disturbed F-actin organization in murine SCs overexpressing miR-181c/d or silencing *Pafah1b1*.

Previous reports have shown that PAFAH1B1 can promote CDC42 activation possibly through interacting with IQGAP1, thereby regulating F-actin cytoskeleton [50]. We then predicted PAFAH1B1 directly interacted with IQGAP1 using the ZDOCK server (Fig. 6i). Co-immunoprecipitation assay in murine SCs further demonstrated the interaction between PAFAH1B1 and IQGAP1 (Fig. 6j, k). In addition, knockdown of *Pafah1b1* or overexpression of miR-181c/d reduced the PAFAH1B1-IQGAP1 complex (Fig. 6l, m). Therefore, decreased PAFAH1B1-IQGAP1 complex downregulates the expression levels of CDC42 and downstream actin-regulatory proteins.

## Discussion

The abnormal number and/or function of Sertoli cells can cause impaired spermatogenesis and male sterility ultimately [51, 52]. Our present results show that overexpression of miR-181c/d perturbs the Sertoli cell barrier in vitro and in vivo, which may be mediated by destabilizing the attachment site between BTB-associated proteins and F-actin. Meanwhile, miR-181c/d suppresses the proliferation and induces the apoptosis of Sertoli cells. Mechanically, miR-181c/d negatively regulates *Pafah1b1* and reduces the PAFAH1B1-IQGAP1 complex. The decreased PAFAH1B1-IQGAP1 complex downregulates the expression levels of CDC42, which leads to the alterations in F-actin organization by inhibiting CDC42 downstream PAK1, LIMK1, and p-Cofilin (Fig. 6n). The results indicate that miR-181c/d acts as a key regulator of Sertoli cell survival and barrier function, thereby affecting the spermatogenesis process in mammals.

The BTB is composed of basal ES and other junctions between adjacent SCs and is essential for preleptotene spermatocyte transition from the basal to the apical compartment [6, 53]. A previous report has shown that deletion of DICER in differentiated male germ cells results in the disorganization of the cell–cell junctions in the seminiferous epithelium [54]. And changes in the distribution of BTB-associated proteins disrupted BTB function [32, 55]. Similarly, a report indicates that miRNAs and their target genes can manipulate the permeability of blood-tissue barriers [56], by altering the distribution of junction proteins, such as ZO-1, Occludin, and Claudin-5 [57]. In this study, we showed that the distribution of BTB-associated proteins is altered in miR-181c/d mimics transfected SCs and LV-miR-181c/d treated mice. This alteration may be responsible for the disruption of the Sertoli cell barrier/BTB and the increase of abnormal spermatozoa rate. Additionally, similar results are observed in *Pafah1b1* inhibited murine SCs, indicating miR-181c/d regulates the Sertoli cell barrier function possibly via targeting *Pafah1b1*.

F-actin serves as attachment sites for TJ proteins and basal ES proteins in the testis [58] and the alteration in F-actin organization is critical for BTB assembly [59]. On the other hand, the *Pafah1b1* gene is related to intracellular regulators of the actin cytoskeleton by complexing with scaffold protein IQGAP1 and activating *Cdc42* gene [50]. Consistent with these reports, we found that PAFAH1B1 interacts with IQGAP1, and knockdown of *Pafah1b1* significantly decreases CDC42 level in murine SCs. Conditional deletion of *Cdc42* in Sertoli cells leads to the disrupted Sertoli cell polarity and the perturbed BTB function in adult male mice [60], and inactivation of the CDC42 signaling pathway attenuates the endothelial barrier function in mouse lungs [61]. Our study demonstrates that inhibition of *Pafah1b1* results in downregulation of CDC42 and its downstream PAK1, LIMK1, and p-Cofilin. PAK1 regulates Cofilin phosphorylation and affects the actin cytoskeleton by activating LIMK1 [62]. Phosphorylated Cofilin can stabilize the actin cytoskeleton in migrating neurons [63]. Changes in these proteins might lead to improper organization of F-actin in murine SCs, which possibly results in the disruption of the Sertoli cell barrier function. Furthermore, we found that knockdown of miR-181c/d increased actin-regulatory proteins expression, whereas transfection of *Pafah1b1* siRNA or *Cdc42* siRNA partially restored the elevated actin-regulatory proteins levels. Therefore, we reveal a mechanism by which miR-181c/d can affect F-actin organization and the Sertoli cell barrier function via the *Pafah1b1* gene.

Lentiviral vector is a tool for transferring exogenous genes into the testes, which assists us to make further investigation into the spermatogenesis process in mice and rats [64, 65]. There are multiple layers of germ cells and tight junctions between the Sertoli cells in mature seminiferous tubules, while there is only one layer of spermatogonia and no tight junctions in the immature testis [66]. In wild-type mice at age of 15 days, biotin tracer can penetrate the seminiferous tubules, indicating that BTB has not yet fully formed [34]. Accordingly, immature testes were selected for injection, which allow lentiviral vectors enter into the seminiferous tubules to infect cells. In addition, the period of 2-week treatment was chosen based on previous studies [6], to ensure that the lentiviral miRNAs have sufficient time to exert the effects in the testis. In our study, LV-miR-181c/d administration in testes successfully overexpresses miR-181c/d in vivo. As expected, the *Pafah1b1* gene is downregulated and BTB function is perturbed in LV-miR-181c/d treated mice. Additionally, LV-miR-181c/d administration does not induce any changes in testis size and weight, testis and epididymis structure, which may be attributable to the complexity of testicular structure or the limited access of the LV-miR-181c/d to all tubules. Thus, improved delivery methods such as PolyPlus in vivo-jetPEI [67] and adeno-associated viruses (AAVs) transduction [68] are under development. Interestingly, the perturbed BTB and the increased abnormal sperm rate in LV-miR-181c/d injected testes are restored at 6 weeks post the final administration. This recovery may be due to the reduced level of lentiviral-induced miR-181c/d in testes over time.

Increasing amounts of evidence indicate that miRNAs play critical roles in regulating Sertoli cell survival during spermatogenesis [69, 70]. The Sertoli cell number in seminiferous tubules determines the production of germ cells [71]. Dysregulated expression of miR-181 affects cell proliferation and apoptosis in chondrocytes [72] and glioblastoma cells [73]. In line with the findings in the above studies, we found that overexpression of miR-181c/d suppresses proliferation and promotes apoptosis of Sertoli cells. As previously noted, the abnormal apoptosis of Sertoli cells disrupts the BTB function in murine testis [74]. In addition, miRNAs may lead to blood-tissue barrier dysfunction via regulating gene expression at transcriptional and post-transcriptional levels [75]. Therefore, we believe that miR-181c/d promotes the apoptosis of Sertoli cells, which may also affect the formation of the testicular BTB to some extent. On the other hand, in mouse neuroepithelial stem cells, *Pafah1b1* silencing reduces proliferation and increases apoptosis [76]. Similarly, knockdown of *Pafah1b1* also inhibits proliferation and promotes apoptosis of Sertoli cells. Further investigations showed that the regulatory effects of miR-181c/d on Sertoli cell proliferation and apoptosis are partially mediated by the *Pafah1b1* gene. Therefore, miR-181c/d regulates proliferation and apoptosis of Sertoli cells through its target gene *Pafah1b1*. Furthermore, mitochondria-dependent cell apoptosis pathway is modulated by miR-181 via the Bcl-2 protein family [77, 78]. miR-181 can target the 3’ UTRs of anti-apoptotic Bcl-2 family members such as Mcl-1, Bcl-2-L11/Bim, and Bcl-2 [78], which triggers apoptosis through interacting with pro-apoptotic proteins such as Bax and Bak [79]. Likewise, knockdown of *Pafah1b1* disrupts the dynamic formation of the microtubule network, which leads to activate the intrinsic mitochondrial apoptotic pathway [80, 81]. Here, we showed that miR-181c/d can target and regulate PAFAH1B1 and observed that the pro-apoptotic role of miR-181c/d on Sertoli cells can be suppressed by *Pafah1b1*. Therefore, we hypothesize that miR-181c/d regulates cell apoptosis at least mediated through *Pafah1b1*.

In conclusion, miR-181c/d regulates Sertoli cell survival and perturbs the Sertoli cell barrier by targeting the *Pafah1b1* gene. And this interruption of barrier function is achieved by changing the localization pattern of BTB-associated proteins at the Sertoli cell–cell interface and disturbing F-actin organization. Mechanically, the miR-181c/d-*Pafah1b1* axis participates in regulating F-actin organization by inactivating CDC42/PAK1/LIMK1/Cofilin pathway. These findings may help us to better understand the role of miRNAs in mammalian spermatogenesis, and suggest that dysregulated expression of miR-181c/d may be an important indicator for male subfertility or infertility. Hence, manipulation of miR-181c/d expression in vivo or in vitro may contribute to the diagnostic and therapeutic strategies for male subfertility or infertility.

## Supplementary Information

Below is the link to the electronic supplementary material.

Supplementary file1 (PDF 3134 kb)

## Data Availability

The data that support the findings of this study are available from the corresponding author upon reasonable request.

## Abbreviations

BTB: Blood-testis barrier
SC: Sertoli cell
ST: Swine testicular
PAFAH1B1: Platelet-activating factor acetylhydrolase 1b regulatory subunit 1
TEM: Transmission electron microscopy
PBS: Phosphate-buffered saline
DAPI: 4’, 6-Diamidino-2-phenylindole
TJ: Tight junction
TER: Trans-epithelial resistance
Na-F: Sodium fluorescein
CCK-8: Cell Counting Kit-8
PCNA: Proliferating cell nuclear antigen
BCL2: B-cell lymphoma 2
BAX: Bcl-2 associated X protein
3’UTR: 3’untranslated region
RT-qPCR: Real-time quantitative PCR
ES: Ectoplasmic specialization
CDC42: Cell division control protein 42 homolog
PAK1: P21(RAC1) activated kinase 1
LIMK1: LIM domain kinase 1
IQGAP1: IQ motif-containing GTPase activating protein 1
