# Supplementary material for: Sertoli cell survival and barrier function are regulated by miR-181c/d-Pafah1b1 axis during mammalian spermatogenesis
Source: Cell Mol Life Sci. 2022 Aug 25;79(9):498. doi: 10.1007/s00018-022-04521-w (PMC9411099; doi:10.1007/s00018-022-04521-w)
Supplement: Supplementary file 1 — Supplementary file1 (PDF 3134 kb) [file 18_2022_4521_MOESM1_ESM.pdf]

Supplemental Appendix

Sertoli cell survival and barrier function are regulated by miR-181c/d-*Pafah1b1* axis during mammalian spermatogenesis

Yue Feng[1], Dake Chen[1], Tiansu Wang[1], Jiawei Zhou[1,2], Wenning Xu[1], Hao Xiong[1], Rong Bai[1], Shang Wu[1], Jialian Li[1], Fenge Li[1, 3*]

[1]Key Laboratory of Pig Genetics and Breeding of Ministry of Agriculture & Key Laboratory of Agricultural Animal Genetics, Breeding and Reproduction of Ministry of Education, Huazhong Agricultural University, Wuhan 430070, PR China

[2] Institute of Animal Science and Veterinary Medicine, Hubei Academy of Agricultural Sciences, Wuhan 430064, China

[3]The Cooperative Innovation Center for Sustainable Pig Production, Wuhan 430070, PR China

*Correspondence: Dr. Fenge Li. Affiliation: College of Animal Science and Technology, Huazhong Agricultural University, Wuhan, 430070, PR China; ORCID: 0000-0003-3862-9114. Tel: 0086-27-87282091; Fax: 0086-27-87280408; E-mail: lifener@mail.hzau.edu.cn

Supplementary information

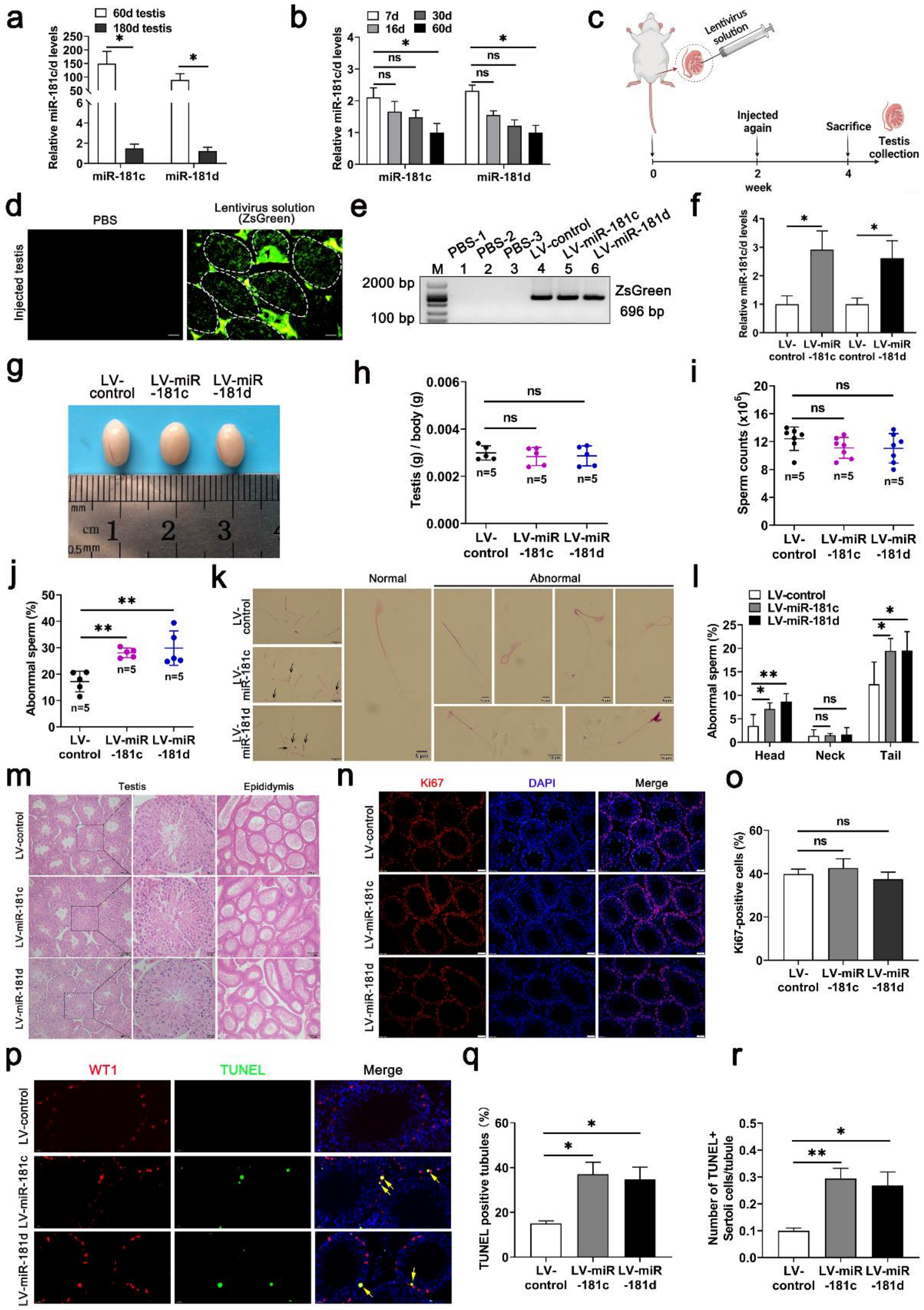

Fig. S1 LV-miR-181c/d administration in testes increases the abnormal sperm rate and cell apoptosis.

Mice were analyzed at two weeks post the final LV-miR-181c/d administration. **a** RT-qPCR analysis of miR-181c/d levels in 60 d and 180 d porcine testis tissues. **b** RT-qPCR analysis of miR-181c/d levels in

testicular tissues of mice at different developmental stages**.** **c** Schematic picture showing the intratesticular injection of lentivirus solution. **d** *In-vivo* transduction efficiency of lentivirus particles. PBS served as a negative control. **e** Semi-quantitative RT-PCR analysis of ZsGreen expression in murine testes injected with PBS and lentivirus solution. PBS served as a negative control. **f** RT-qPCR analysis of miR-181c/d levels in the LV-control mice and the LV-miR-181c/d mice. **g-i** The testis size (**g**) ($n = 5$), the ratio of testis weight to body weight (**h**) ($n = 5$), and the sperm counts (**i**) ($n = 5$). **j** The abnormal sperm rate in the LV-control mice and the LV-miR-181c/d mice. **k** Sperm morphology was detected by Giemsa staining ($n = 5$). Black arrows indicate abnormal sperm. **l** The rates of head, neck, and tail deformed sperm. **m** The representative images of testicular (left panel) and epididymis (right panel) cross-sections stained with hematoxylin and eosin ($n = 3$). Scale bars: 20 and 100 μm. **n** Immunofluorescence staining of the cell proliferation marker Ki67 (red) in testes ($n = 3$). Scale bar: 50 μm. **o** Quantification of Ki67-positive cells. **p** Representative images of TUNEL (green) and WT1 (red) double immunofluorescence staining of testes sections ($n = 3$). TUNEL/WT1 double-positive cells are indicated by yellow arrows. Scale bar: 20 μm. **q** Quantification of the numbers of TUNEL-positive seminiferous tubules. **r** Quantification of the numbers of TUNEL-positive Sertoli cells per seminiferous tubule. Data are presented as mean ± SD of at least three independent experiments. $*p < 0.05$; $**p < 0.01$; ns, not significant

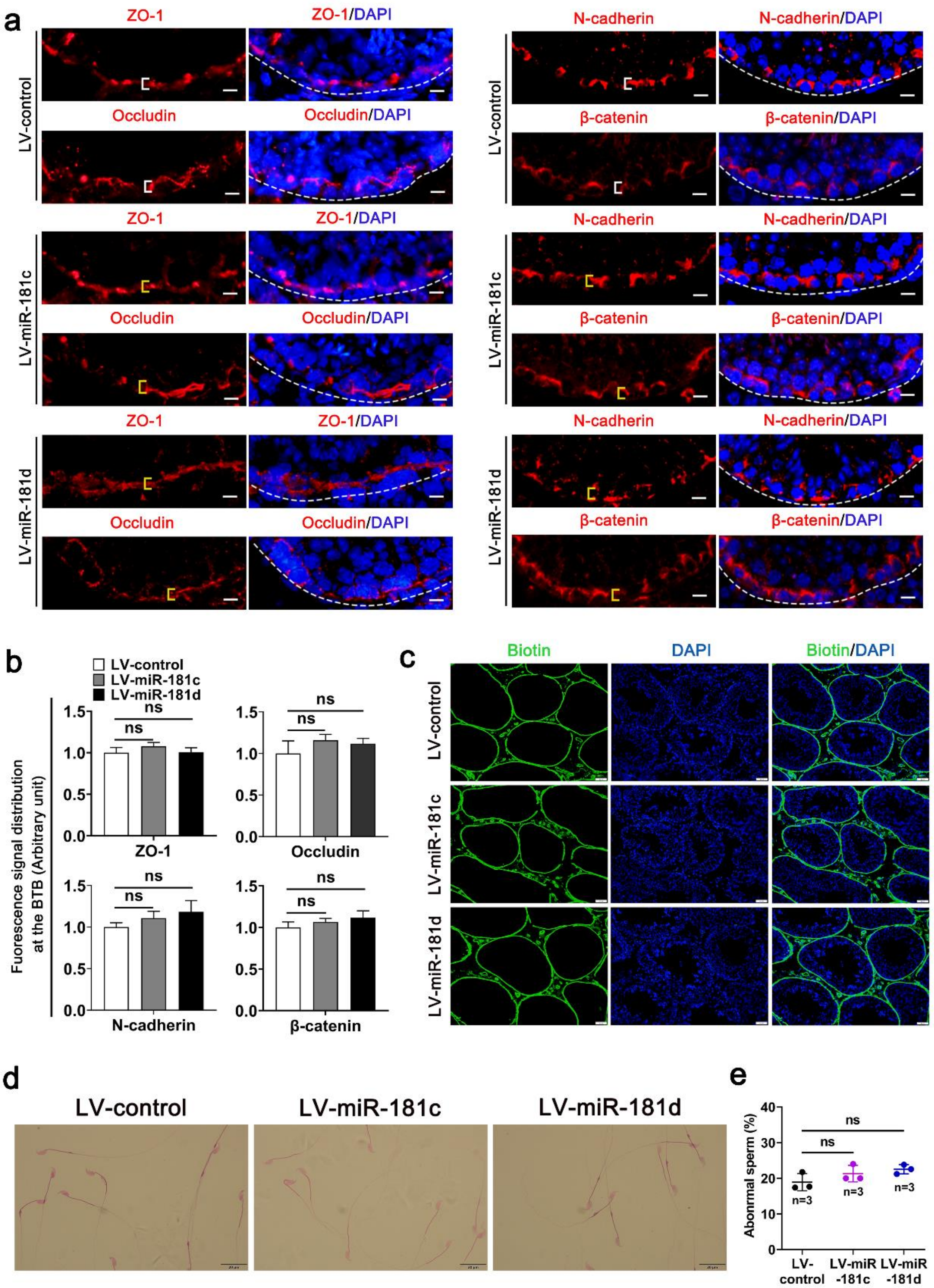

Fig. S2 Disturbed BTB function induced by LV-miR-181c/d administration in testes is not permanent.

Mice were analyzed at six weeks post the final LV-miR-181c/d administration. **a** Immunofluorescence staining of TJ proteins (red) and basal ES proteins (red) in testes (n = 3). These proteins are tightly localized at the BTB near the basement membrane (dashed white line) in both the LV-control mice (white

bracket) and the LV-miR-181c/d mice (yellow brackets). Scale bar: 10 µm. **b** Relative quantification of fluorescence signal distributed at the BTB. **c** *In vivo* BTB integrity assay (n = 3). Scale bar: 50 µm. **d** Sperm morphology was detected by Giemsa staining (n = 3). **e** The abnormal sperm rate in the LV-control mice and the LV-miR-181c/d mice at six weeks post the final LV-miR-181c/d administration. Data are presented as mean ± SD (n = 3). ns, not significant

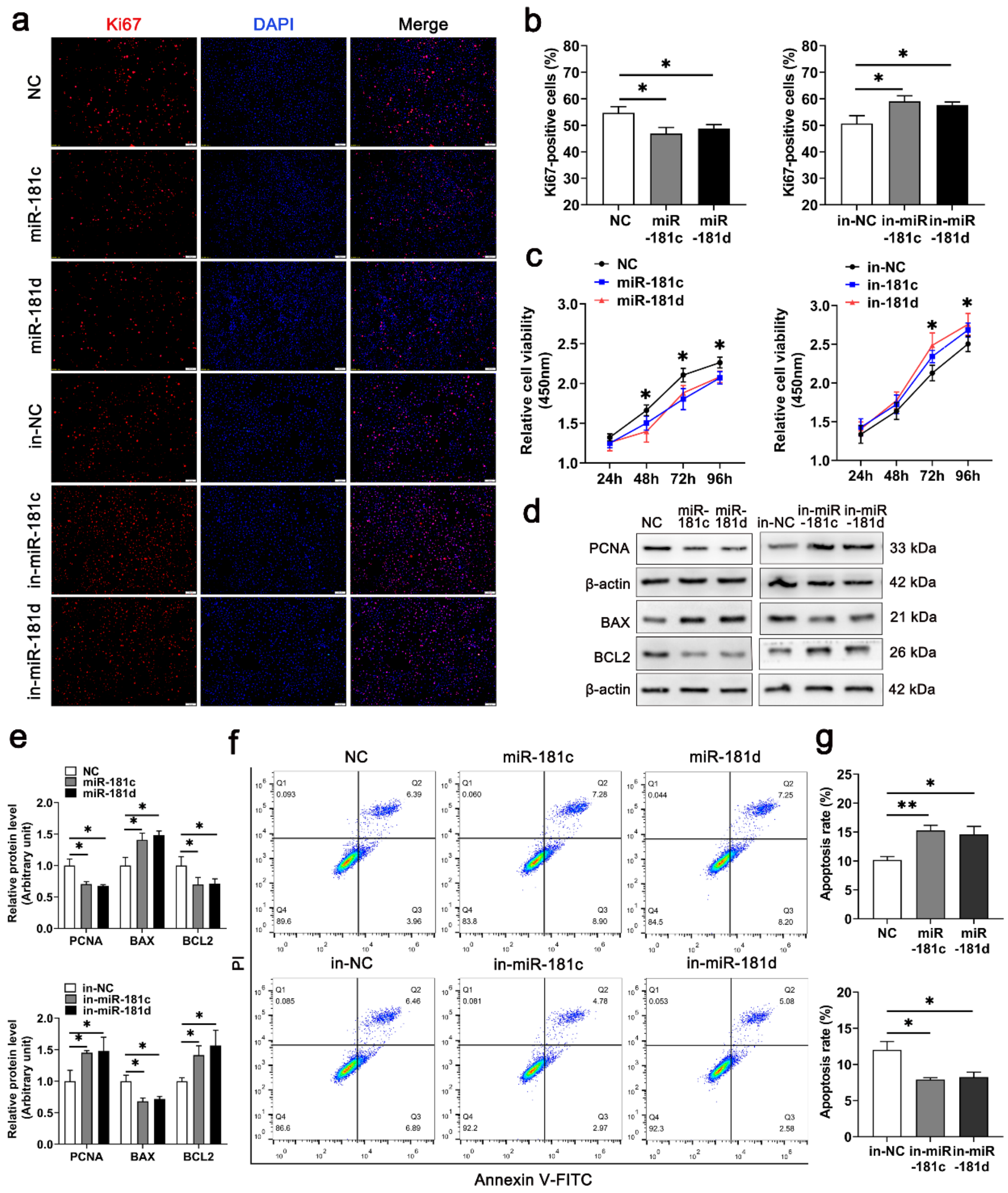

Fig. S3 miR-181c/d inhibits proliferation and promotes apoptosis of porcine ST cells.

The porcine ST cells were transfected with mimics NC, miR-181c/d mimics, inhibitors NC, or miR-181c/d inhibitors. miR-181c mimics, miR-181d mimics, and mimics NC are abbreviated to miR-181c, miR-181d, and NC, respectively. miR-181c inhibitors, miR-181d inhibitors, and inhibitors NC are abbreviated to in-miR-181c, in-miR-181d, and in-NC, respectively. **a** Immunofluorescence staining of Ki67 (red) in miR-181c/d mimics or inhibitors treated porcine ST cells. Scale bar: 100 μm. **b** Quantification of Ki67-positive cells in miR-181c/d mimics or inhibitors treated porcine ST cells. **c** CCK-8 assay performed in miR-181c/d mimics or inhibitors treated porcine ST cells. **d** Western blot analysis

of PCNA, BAX, and BCL2 in miR-181c/d mimics or inhibitors treated porcine ST cells. The quantification of protein level is shown in the bar graph (**e**). **f** Annexin V-FITC/PI and flow cytometry analysis was used to examine cell apoptotic rate in miR-181c/d mimics or inhibitors treated porcine ST cells. **g** Quantification of cell apoptotic rate in miR-181c/d mimics or inhibitors treated porcine ST cells. Data are presented as mean ± SD of at least three independent experiments. $^{*}p < 0.05$; $^{**}p < 0.01$

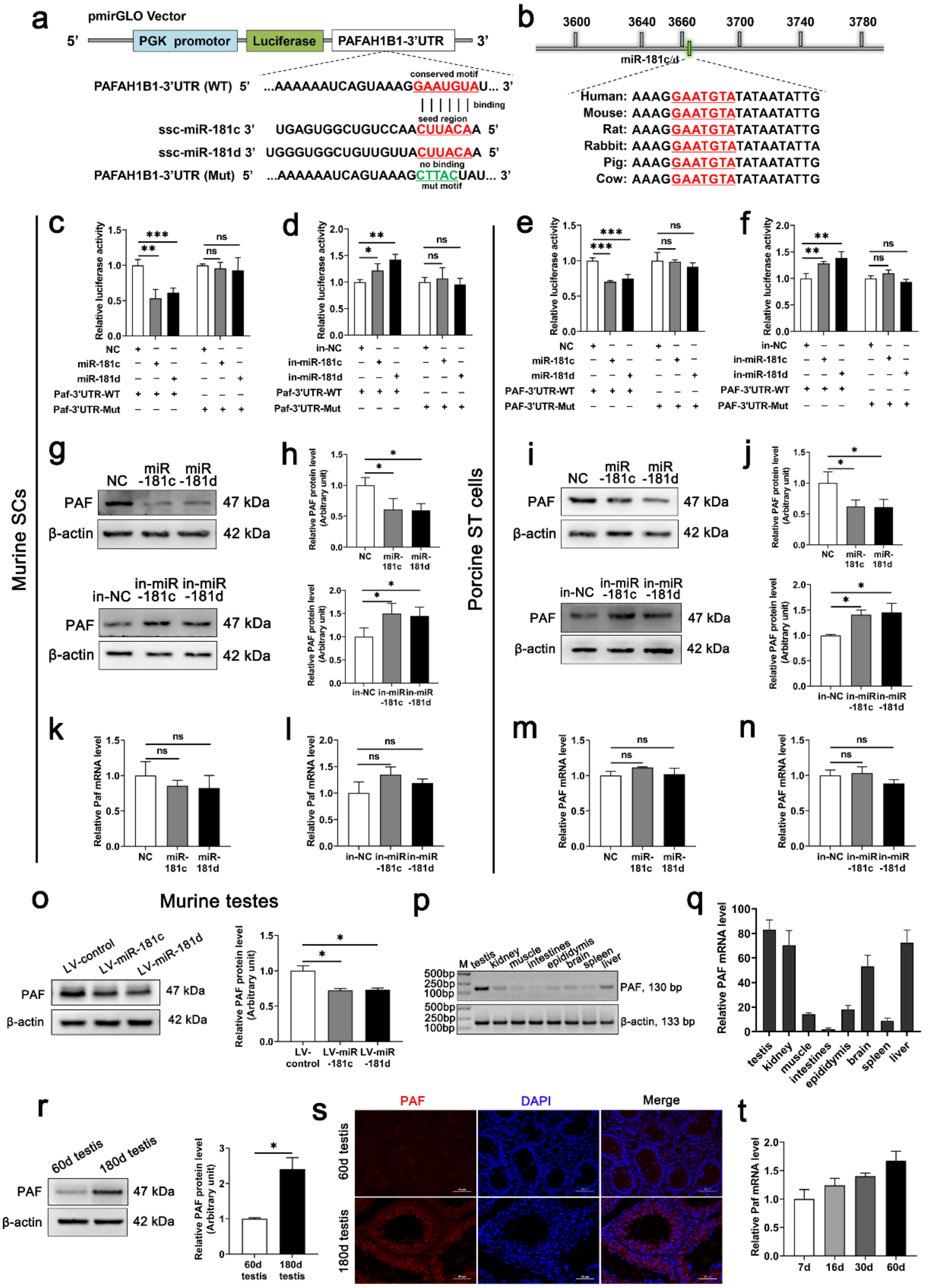

Fig. S4 ***Pafah1b1*** **gene is a target of miR-181c/d in Sertoli cells.**

The Sertoli cells (murine SCs and porcine ST cells) were transfected with mimics NC, miR-181c/d mimics, inhibitors NC, or miR-181c/d inhibitors. Pafah1b1 is abbreviated to Paf; PAFAH1B1 is abbreviated to PAF. **a** Schematic representation of the *PAFAH1B1* 3' UTR and its miR-181c/d-binding sites. **b** The predicted sequences to the seed regions of miR-181c/d within the *PAFAH1B1* 3' UTR are

conserved across species. Red bases indicate the conserved sequence. **c-f** Dual-luciferase reporter assay was performed to confirm the binding of miR-181c/d and *Pafah1b1* 3' UTR in murine SCs (**c, d**) and porcine ST cells (**e, f**). **g-j** Western blot analysis and quantification of PAFAH1B1 protein in murine SCs (**g, h**) or porcine ST cells (**i, j**). **k-n** RT-qPCR analysis of *Pafah1b1* mRNA in murine SCs (**k, l**) or porcine ST cells (**m, n**). **o** Western blot analysis and quantification of PAFAH1B1 protein in LV-miR-181c/d treated murine testes. **p, q** Semi-quantitative RT-PCR (**p**) and RT-qPCR (**q**) analysis of *PAFAH1B1* level in multiple tissues of Large White boars. **r, s** Western blot (**r**) and immunofluorescence staining (**s**) were performed to examine the expression of PAFAH1B1 in testes from 60 d and 180 d Large White boars. Scale bar: 50 μm. **t** RT-qPCR analysis of *Pafah1b1* levels in testicular tissues of mice at different developmental stages**.** Data are presented as mean ± SD of at least three independent experiments. *$p < 0.05$; **$p < 0.01$; ***$p < 0.001$; ns, not significant

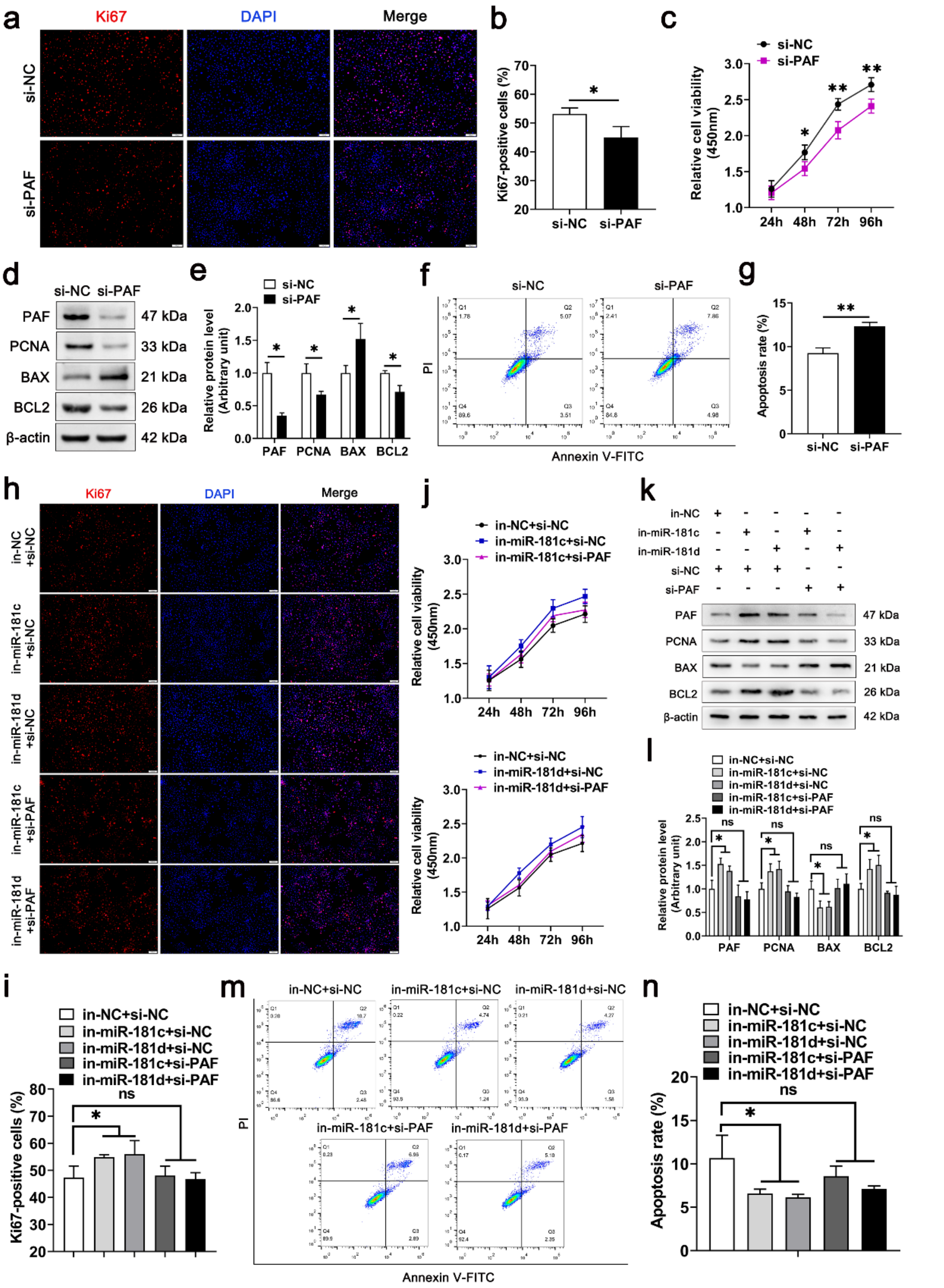

Fig. S5 *PAFAH1B1* knockdown reverses the pro-growth of miR-181c/d inhibited porcine ST cells. The porcine ST cells were transfected with NC siRNA or *PAFAH1B1* siRNA. PAFAH1B1 is abbreviated to PAF. **a** Immunofluorescence staining of Ki67 (red) in *PAFAH1B1* siRNA treated porcine ST cells. Scale bar: 100 μm. **b** Quantification of Ki67-positive cells in *PAFAH1B1* siRNA treated porcine ST cells. **c** CCK-8 assay performed in *PAFAH1B1* siRNA treated porcine ST cells. **d** Western blot analysis of

PAFAH1B1, PCNA, BAX, and BCL2 in *PAFAH1B1* siRNA treated porcine ST cells. The quantification of protein level is shown in the bar graph (**e**). **f** Annexin V-FITC/PI and flow cytometry analysis was used to examine cell apoptotic rate in *PAFAH1B1* siRNA treated porcine ST cells. **g** Quantification of cell apoptotic rate in *PAFAH1B1* siRNA treated porcine ST cells. Five co-transfection treatments were constructed in this experiment, including inhibitors NC + NC siRNA, miR-181c inhibitors + NC siRNA, miR-181d inhibitors + NC siRNA, miR-181c inhibitors + *PAFAH1B1* siRNA, and miR-181d inhibitors + *PAFAH1B1* siRNA. **h-j** Ki67 staining (**h**) and CCK-8 (**j**) assay were performed in porcine ST cells treated with co-transfections. Quantification of Ki67-positive porcine ST cells treated with co-transfections (**i**). Scale bar: 100 µm. **k** Western blot analysis of PAFAH1B1, PCNA, BAX, and BCL2 in porcine ST cells treated with co-transfections. The quantification of protein level is shown in the bar graph (**l**). **m** Annexin V-FITC/PI and flow cytometry analysis was used to examine cell apoptotic rate in porcine ST cells treated with co-transfections. **n** Quantification of cell apoptotic rate in porcine ST cells treated with co-transfections. Data are presented as mean ± SD of at least three independent experiments. *$p < 0.05$; **$p < 0.01$; ns, not significant

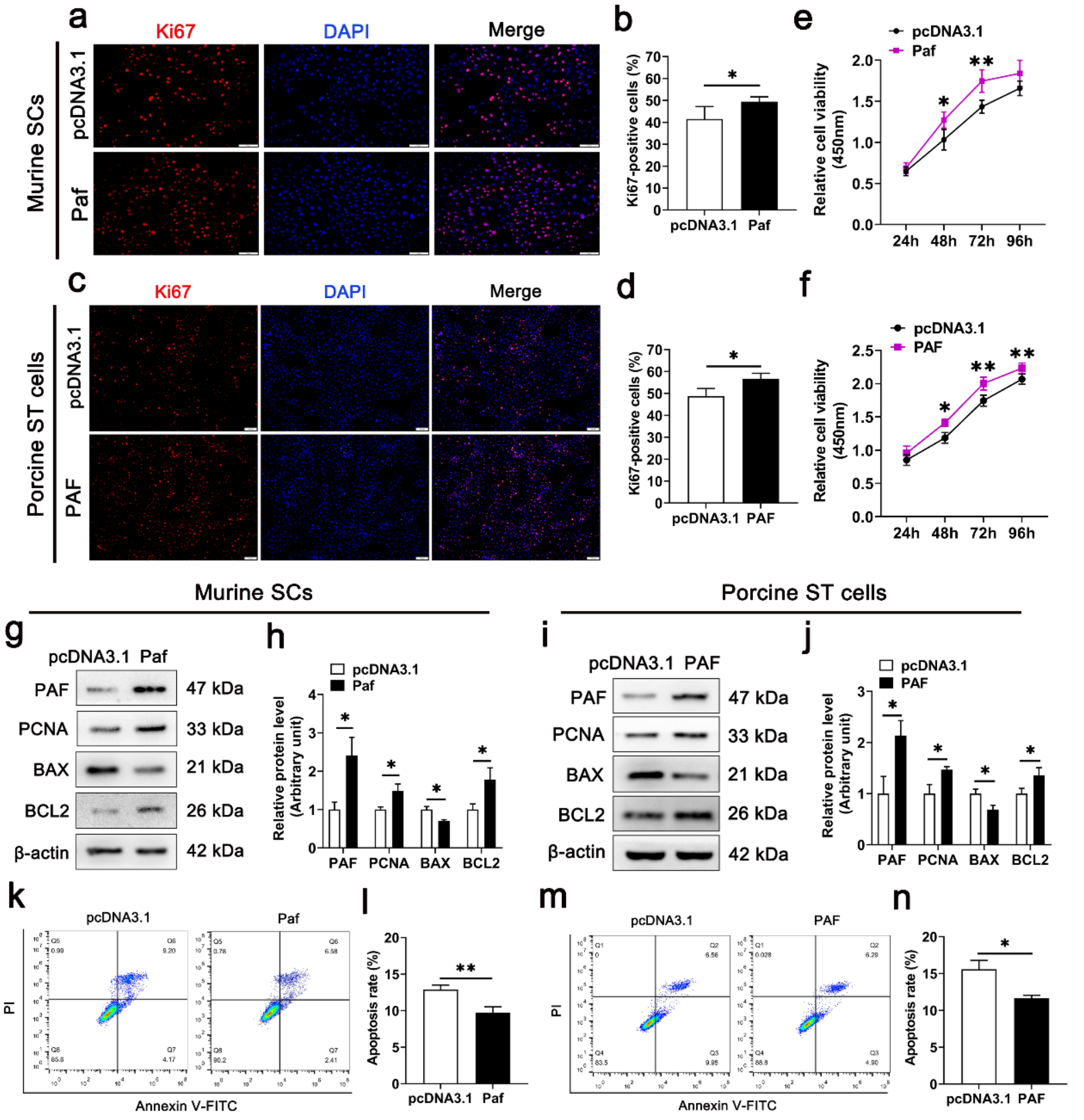

Fig. S6 Overexpression of *Pafah1b1* promotes proliferation and inhibits apoptosis of Sertoli cells. The Sertoli cells (murine SCs and porcine ST cells) were transfected with *pcDNA3.1*, *pcDNA3.1-Pafah1b1*, or *pcDNA3.1-PAFAH1B1*, respectively. *pcDNA3.1-Pafah1b1* is abbreviated to Paf, *pcDNA3.1- PAFAH1B1* is abbreviated to PAF. **a, c** Immunofluorescence staining of Ki67 (red) in murine SCs (**a**) and porcine ST cells (**c**). Scale bar: 100 μm. **b, d** Quantification of Ki67-positive cells. **e, f** CCK-8 assay performed in murine SCs (**e**) and porcine ST cells (**f**). **g-j** Western blot analysis and quantification of PAFAH1B1, PCNA, BAX, and BCL2 protein in murine SCs (**g, h**) or porcine ST cells (**i, j**). **k, m** Annexin V-FITC/PI and flow cytometry analysis was used to examine cell apoptotic rate in murine SCs (**k**) and porcine ST cells (**m**). **l, n** The quantification of cell apoptotic rate. Data are presented as mean ± SD of at least three independent experiments. *$p < 0.05$; **$p < 0.01$
